# Enhancing Biodiversity‐Function Relationships in Field Retting: Towards Key Microbial Indicators for Retting Control

**DOI:** 10.1111/1758-2229.70102

**Published:** 2025-06-18

**Authors:** Eliane Bou Orm, Suvajit Mukherjee, Etienne Rifa, Anne Créach, Sébastien Grec, Sandrine Bayle, Jean‐Charles Benezet, Anne Bergeret, Luc Malhautier

**Affiliations:** ^1^ Polymers, Composites and Hybrids (PCH) IMT Mines Alès Alès France; ^2^ Laboratoire Des Sciences Des Risques (LSR) IMT Mines Alès Alès France; ^3^ Univ Lille, CNRS UMR 8576—UGSF—Unité de Glycobiologie Structurale et Fonctionnelle Lille France; ^4^ Toulouse Biotechnology Institute (TBI), Plateforme Genome et Transcriptome (GeT‐Biopuces), Université de Toulouse, CNRS, INRAE, INSA Toulouse France

**Keywords:** bacterial and fungal communities, enzymatic activity, field retting, hemp, high‐throughput sequencing, metabarcoding, temporal dynamics

## Abstract

Hemp field retting is a bioprocess that facilitates fibre extraction by degrading pectin and other matrix components surrounding fibre bundles. However, traditional methods rely on empirical practices, often resulting in inconsistent fibre quality. This study investigates the biodiversity–function relationship in the hemp retting ecosystem to identify microbial and enzymatic indicators for improved process control. Over six weeks of field retting, we monitored bacterial and fungal community dynamics using high‐throughput sequencing and assessed enzymatic activity profiles. Our results revealed a sequential enzymatic pattern: pectinases (e.g., polygalacturonase) dominated early stages, followed by hemicellulases (β‐xylosidase, β‐galactosidase), and later cellulases. These enzymatic shifts were reflected in the changes in microbial community composition, with pectinolytic bacteria (e.g., *Proteobacteria*) dominating the initial phases and cellulolytic fungi (e.g., *Ascomycota*) becoming more prevalent later. Our results identified specific microbial taxa correlated with optimal retting, suggesting their potential as bioindicators for monitoring retting. Specifically, key bacterial genera such as *Pseudomonas* and *Sphingomonas*, and fungal genera like *Cladosporium*, were associated with distinct enzymatic profiles. Our findings offer new insights into the microbial ecology of retting, providing both microbial and enzymatic indicators that could inform the development of monitoring strategies for process control, ultimately contributing to more consistent hemp fibre production.

## Graphical abstract

This study investigates the temporal dynamics of bacterial and fungal communities involved in hemp field retting. Using high‐throughput sequencing, this study identified key bacterial and fungal phyla in hemp stems and soil samples, including *Proteobacteria*, *Ascomycota*, and newly discovered phyla. Enzymatic activity analysis reveals biological processes crucial to fibre extraction. The findings aim to improve retting control and enhance fibre quality.

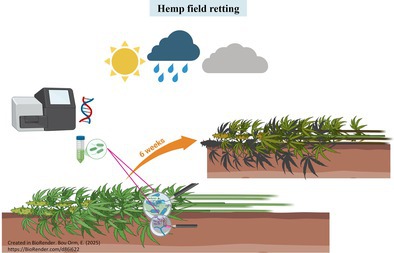

## 1 Introduction

Lignocellulosic biomass, which is the most abundant source of organic matter on Earth (Okolie et al. 2021), has a complex structure and is composed mainly of three polymers (cellulose, hemicelluloses, and lignin). In the context of the transition to a low‐carbon world, materials such as plant fibres (hemp, flax, kenaf, etc.), which are renewable sources of lignocellulosic biomass, are of growing interest (Ten and Vermerris 2013; Rehman et al. 2021). Compared to non‐renewable fibres, that is, glass fibres, plant fibres are moreover interesting for their lower cost and lower density, while still exhibiting challenging specific mechanical properties (properties scaled to density) (Bourmaud et al. 2020; Speight 2020).

Among lignocellulosic plants, hemp plants have recently been attracting ever‐increasing interest worldwide for different reasons. Firstly, climate change (especially drought) strongly impacts flax cultivation and can lead to reduced yields and poor fibre quality (Beauvais et al. 2022; Le Duigou et al. 2011). On the opposite, hemp could resist various climatic conditions and does not necessitate crop rotation or the use of pesticides compared to flax (Amaducci et al. 2015).

For efficient extraction of hemp fibres from stems without altering their ultimate properties, a prior bioprocess called retting must be carried out (Mazian et al. 2020; Réquilé et al. 2021; Zimniewska 2022). This retting process aims to facilitate the subsequent mechanical extraction of plant fibres from the plant stems by ensuring their decohesion (Placet et al. 2017). Currently, field retting is the most prevalent method used in Europe, mainly due to its cost‐effectiveness, ease of implementation, and environmental sustainability. Although other types of retting exist, such as water, chemical, and enzymatic retting, field retting (dew‐retting) is preferred over these methods (Sisti et al. 2018). The field retting process is carried out by the farmers in an empirical way and is largely based on their experience. This biological process consists of laying out the plant stems on the ground and exposing them to local environmental conditions for a few weeks after harvesting. During retting, the decohesion of the fibres is realised as a result of the secretion of enzymes by the microorganisms colonising the stems and those present in the soil (Bleuze et al. 2018). Enzymatic activities ensure the degradation of the parenchyma cells surrounding the fibre bundles, leading to their decohesion from the other components of the stem (Müssig 2010). The main challenge of retting is to facilitate the separation of fibres by dissolving the binding agents between them while preventing excessive retting that may damage the cellulosic fibres (Richely et al. 2022). Several studies have focused on the analysis of the physicochemical, biochemical, and mechanical properties of fibres at the macroscopic level during retting (Placet et al. 2017; Mazian et al. 2019, 2020; Lee et al. 2020). Studies on the microscopic level, particularly concerning the microbial communities involved in the process, are limited. Until now, the microbiological and biochemical mechanisms underlying the field retting of hemp are still poorly documented. A better understanding of the main actors of retting, which are the microbial communities, is then essential for more effective management of this process. The temporal dynamics of the microflora, including enzymatic activities and colonisation dynamics, have only recently been investigated (Ribeiro et al. 2015; Law et al. 2020). While retting offers numerous environmental benefits, the growing industrial application of hemp‐based products raises concerns about the potential environmental risks posed by nanomaterials derived from fibres (Enarevba and Haapala 2024). As these materials are scaled for industrial use, there is a need to address the risks of nanoparticle release into the environment. Nanotoxicity is becoming an emerging concern due to the potential for nanoparticles to interact with the environment and human health (Kumah et al. 2023). Thus, a deeper understanding of microbial mechanisms during retting could provide insights into the potential nanomaterials production from fibres.

The study of retting in other fibre plants has benefited from the application of new meta‐omics approaches based on high‐throughput sequencing techniques. Only three studies used high‐throughput sequencing technologies (Ion Torrent PGM system for bacterial domain) to analyse kenaf retting (Visi et al. 2013), water retting of flax (Zhao et al. 2016), and recently, bacterial and fungal domains on field retting of flax (Illumina MiSeq) (Djemiel et al. 2017), and the bacterial community of kenaf water retting (Illumina MiSeq) (Xu et al. 2022). Most other studies provided only a partial characterisation of the retting microbiota by using both cultured approaches (morphological, metabolic, and physiological characteristics) and phenotypic classifications (Brauman et al. 1996; Moawad et al. 2019; Datta et al. 2020). Ribeiro et al. (2015) conducted research on the microbiology of hemp field retting using sequence analysis of randomly cloned 16S and 18S ribosomal RNA (rRNA) genes. However, this method has limitations in detecting the total microbial communities (Sahoo et al. 2017). Law et al. (2020) focused solely on the bacterial community of controlled hemp retting using the16S rRNA gene amplicon sequencing (Illumina MiSeq platform).

This study uses high‐throughput sequencing to comprehensively characterise the bacterial and fungal communities involved in the hemp retting process. This approach offers a deeper understanding of microbial diversity and enzymatic potential compared to previous studies relying on partial characterisation or cultured techniques.

This study addresses three main objectives: (1) to characterise temporal dynamics of bacterial and fungal communities during hemp field retting using high‐throughput sequencing; (2) to evaluate key enzymatic activities associated with progressive degradation of plant cell wall components; and (3) to identify specific microbial taxa and functional indicators predictive of optimal fibre separation efficiency.

## 2 Experimental Procedures

### 2.1 Hemp Retting

#### 2.1.1 In the Field

Hemp plants ( *Cannabis sativa* L., Cultivar Futura 75) were sown at a rate of 40 kg/ha on April 15, 2021, in the south of France in the Drôme Chanvre association (http://www.dromechanvre.fr/) (Mirabel et Blacons, France, 44°42′35″ N, 5°05′29″ E). The hemp plants were harvested at the end of flowering (September 2, 2021) using a sectional mower adapted to this crop. After harvest, hemp plants were transported to another field in the south of France named Mas de La Valus (Le Bouquet, France, 44°09′52′′ N, 4°16′56′′ E) for easy logistics (close to the laboratory) and retted in this field (Figure S1) (Bou Orm et al. 2024a).

Hemp plants were retted from September 3, 2021, until October 17, 2021. The swaths were defined according to a non‐systematic W pattern shown in Figure S2. Five swaths (250 stems/swath) were retted according to the weather conditions. Hemp stems were turned regularly (once a week) to homogenise the retting of the stems (Djemiel et al. 2017).

#### 2.1.2 In a Retting Pilot Unit

The retting process was carried out at the laboratory by using a pilot unit containing the soil from the cultivation plot (Mirabel et Blacons, France). The plants were grown and retted on typical clayey gravelly soil with a slightly basic pH (8.1 at Mirabel et Blacons and 8.4 at Mas de la Valus). The polypropylene‐made pilot unit measures 1 m in length, 1 m in width, and 25 cm in height. It is filled with gravel/pozzolan (10 cm high) and contains 15 cm of soil recovered directly from the hemp field (Figure S3). A swath of 250 stems of Futura 75 was placed in the pilot unit, and the stems were turned once a week.

### 2.2 Sample Collection

Throughout the study, samples of stems and soil were collected weekly from five swaths in the field and the pilot unit at regular intervals (Figure S4). The collected samples (20 stems/week) included unretted stems (R0), retted stems for 1 (R1), 2 (R2), 3 (R3), 4 (R4), and over‐retted stems for 6 (R6) weeks, respectively. Soil samples were also collected weekly from both the field and the pilot unit retting. However, in this study, only soil samples obtained at R0, R2, and R4 from the field, and R0 and R2 from the pilot unit, were considered. It has been exhibited (Djemiel et al. 2017) that the microbial composition of soil remains slightly constant during retting. Stem samples were immediately stored at −80°C, while soil samples were collected at a depth of 20 cm, sieved (with a pore size less than 1.0 mm), freeze‐dried, and stored at −80°C for further analysis.

### 2.3 DNA Extraction and Purification

The DNA extraction procedure followed the Genosol platform protocol (INRAe, Dijon, France) with an internal reference of G.MO‐026.6 from 2020 (Lelievre 2020; Bou Orm et al. 2023, 2024a). To perform cell lysis, each sample (1 g of soil and 2 g of stem) was shaken in the FastPrepR‐24 in 15 mL Falcon tubes with 2 g of ceramic, silica, and glass beads and 5 mL of lysis buffer containing EDTA (100 mM, pH 8), Tris (100 mM, pH 8), NaCl (100 mM) and SDS (2%). The samples were incubated at 70°C with agitation at 300 rpm for 30 min to perform mechanical and chemical lysis. After incubation, the samples were centrifuged at 7000 g for 5 min, and 1 mL of the lysate was collected. To remove proteins, 100 μL of potassium acetate (3 M, pH 5.5) was added to the lysate, and the supernatant was collected after centrifugation at 14,000 g for 5 min. To precipitate the DNA, 900 μL of isopropanol (−20°C) was added to the samples, which were then incubated at −20°C for 1 h (for soil) or 30 min (for stems). After centrifugation at 16,200 g for 30 min, the supernatant was removed. The DNA pellet was washed with 400 μL of 70% ethanol (−20°C), centrifuged (16,200 g, 2 min), and dried at 60°C for 10–15 min. The crude DNA pellet was resuspended in 200 μL of ultrapure water after 4 h of storage at 4°C and stored at −20°C.

The DNA was extracted from stems (3 replicates or swaths per week, and 1 replicate per week of field and pilot‐unit retting respectively) and soil (3 replicates or swaths per week and 1 replicate per week of field and pilot‐unit retting respectively). These extracted DNA samples are then purified using the Nucleospin Soil Kit (Macherey Nagel, Düren, Germany) in two steps. The first step involves molecular sieving on a NucleoSpin Inhibitor Removal Column, and the second step involves DNA binding to a silica membrane using a NucleoSpin Soil Column. The purification is carried out in accordance with the manufacturer's guidelines. Following purification, a total volume of 80 μL of DNA is obtained, which is used as a template for further analyses.

### 2.4 DNA Quantification

The quantity of extracted DNA was initially determined by Nanodrop technology, by using 1.5 μL of extracted DNA and measuring absorbance at 260 nm. The extracted DNA was then further quantified with greater accuracy by measuring absorbance at 535 nm following PicoGreen staining using the Quant‐iT PicoGreen dsDNA Assay kit (Thermo Fisher, Bleiswijk, Netherlands) (Bou Orm et al. 2023). This accurate quantification was used to assess the abundance of bacterial and fungal communities through qPCR (quantitative polymerase chain reaction) and for taxonomic composition (Bou Orm et al. 2024a).

The purity of DNA samples was assessed using a NanoDrop spectrophotometer (ThermoScientific, Wilmington, USA) by measuring the absorbance of 1.5 μL of DNA at 280 and 230 nm. The ratios of the 260/280 nm and 260/230 nm values were calculated to determine the purity. The absorbance at 280 nm primarily represented protein contamination, while the absorbance at 230 nm was indicative of the presence of organic compounds, proteins, or chaotropic substances (Bou Orm et al. 2024a).

### 2.5 High‐Throughput Sequencing

Multiplex sequencing was carried out with Illumina Mi‐Seq 2 × 250 bp technology on the GeT‐Biopuces platform (Toulouse Biotechnology Institute, INSA, Toulouse, France) (http://get.genotoul.fr/). The PCR amplifications of the V3‐V4 region of the 16S rRNA gene (for bacteria) and the V5 region of the 18S rRNA gene (for fungi) were performed (Table S1). The first step was the amplification (PCR1) of the region of interest using fusion primers containing half the sequence of Illumina adapters and the specific sequence of the interest region. After the bead's purification, the quality (length, quantity) was checked using the DNA 12,000 assay (Agilent 2100 Bioanalyzer, Santa Clara, CA, USA) and nanodrop ND‐8000 (Thermofisher, Wilmington, USA). Magnetic beads were used in sequencing to isolate and purify DNA by removing residues. The addition of the complete Illumina adapters (P5/P7) and the barcoding (6 bp unique on the P7 adapter) of each sample were made by PCR (PCR2). After beads purification and quality control as after PCR1, the same quantity of each sample was pooled and loaded onto the Illumina MiSeq cartridge (MiSeq Reagent Kit v3 (600 cycles), California, USA) according to the manufacturer's instructions. The quality of the run was checked internally using PhiX (PhiX Control v3, California, USA), and then, each pair‐end sequence was assigned to its sample with the help of the previously integrated index. The PhiX internal control is a quality DNA commonly used in sequencing analysis to ensure that the data produced is of high quality and unbiased.

Microbial data sets supporting the results in this article are available at NCBI with accession number PRJNA973023.

### 2.6 Sequence Analysis

The analysis of targeted metagenomic data was performed using the R package rANOMALY (Theil and Rifa 2021). For each marker (16S V3V4 and 18S V5), the bioinformatic workflow began by processing the raw data (fastq files) and applying filters on sequences missing exact primers and low‐quality sequences. The rANOMALY workflow generates a raw abundance table of sequence variants using the denoising method from the DADA2 package (Callahan et al. 2016). Chimeric amplicon sequence variants (ASV) are filtered out before downstream analysis. Then, taxonomic assignment is carried out for each sequence variant using the IDTAXA algorithm (Murali et al. 2018) against GTDB (Parks et al. 2022) and SILVA 138 databases (Quast et al. 2013). Abundance table, taxonomy table, representative sequences, and samples metadata were aggregated into a phyloseq object for statistical analysis (McMurdie and Holmes 2013). Various functions of rANOMALY and Explore Metabar 2.0.1 (https://explore‐metabar.sk8.inrae.fr/) (Rifa and Theil 2010) were then utilised for statistical analyses, including alpha and beta diversity analyses and visualisation of the microbial community.

Alpha diversity was evaluated using non‐parametric estimators such as the Chao1 index and Pielou's evenness index. Microbial diversity was estimated using Shannon's and inverse Simpson's indexes.

Alpha diversity determines the microbial community's specific richness in each sample. The specific richness represents the number of ASV (Amplicon Sequence Variant) present in each sample. Chao1 value provides an estimate of the total species richness in the sample, including unobserved species (O'Hara 2005; Eren et al. 2012). The Shannon index (H′) considers both the richness and relative abundance of each ASV to assess the degree of microbial community equilibrium. For an equal number of ASV, a balanced community has a higher Shannon index than a community characterised by a few dominant taxa compared to the rest of the community. The Shannon index is calculated using the following Equation (1):

(1) $$H^{\prime }=-\sum \left(\mathrm{pi}\times \ln\;\left(\mathrm{pi}\right)\right)$$

with, *p*_*i*_ representing the proportion of individuals belonging to the *i*‐th species (***p***_***i***_ = $\frac{\mathrm{ni}}{N}$, where *n*_*i*_ is the population size of species *i* and *N* is the total number of individuals in the community).

Since the Shannon index depends on both the relative abundance and the number of ASV, it is usually supplemented by the Pielou index. The Pielou index measures equitability in addition to species diversity because it measures both species richness and evenness. Pielou index is calculated using the Equation (2):

(2) $$J=\frac{H^{\prime }}{H\max}=\frac{H^{\prime }}{\ln S}$$

with *H*′ corresponding to the observed diversity, Hmax is the maximum theoretical diversity and *S* is the total number of species present. The Pielou index varies between 0 and 1. When species have identical abundances, the Pielou index value is close to 1, and it is minimal when one species dominates the entire population. Maximum diversity is achieved when species have an equitable distribution (Marcon 2010).

The Shannon index gives the same importance to all individuals. However, the inverse Simpson index gives less importance to rare species (Simpson 1949; Schlaepfer and Bütler 2002; Marcon 2010).

Beta diversity, reflecting variations in microbial composition and membership across samples, was analysed using a Bray‐Curtis distance matrix. Non‐Metric Multidimensional Scaling (NMDS) and Principal Coordinate Analysis (PCoA) based on the Bray‐Curtis index were used to visualise the similarities and dissimilarities between samples.

To assess the statistical significance of dissimilarities in the microbial community structure among multiple samples, in the NMDS analysis, a PERMANOVA with Adonis function from the vegan package (permutation‐based multivariable analysis of variance) is performed. The test is carried out on the distance matrix between the samples calculated from the ASV normalised abundance table (Total Sum Scaling).

The PICRUSt2 (https://github.com/picrust/picrust2) pipeline (Douglas et al. 2020) was used to predict the functional composition of bacterial enzymatic activity abundance using 16S rDNA datasets. The raw ASV abundance table from the rANOMALY workflow and the ASV representative sequences were used in the PICRUSt2 metagenome pipeline function. This workflow reassigns ASV by using a reference tree and multiple sequence alignment. The NSTI (Nearest Sequenced Taxon Index) was calculated, and sequences with NSTI values above two have been excluded. The enzyme abundance results from the multiplication of the 16S copy number normalised ASV abundance and the predicted gene copy number per ASV. The inferred raw abundance of enzymes (EC numbers table) was transformed into relative abundance with TSS normalisation (Total Sum Scaling) for comparison between conditions.

### 2.7 Enzymatic Activities

Stem samples from four retting stages (R0, R1, R4, and R6) have been analysed. The analysed enzymes were peroxidase, phenoloxidase, ß‐D‐galactosidase, ß‐D‐glucosidase, ß‐D‐xylosidase, cellobiohydrolase, and polygalacturonase. These enzymes were selected based on literal data, which are found to be most responsible for wood decomposition, and the measurement of enzymatic activities followed the protocols adapted and modified from previous studies (Bell et al. 2013; Sauvadet et al. 2016; Bleuze et al. 2018; Chabbert et al. 2020; Mukherjee et al. 2024).

After removing the woody xylem part, frozen stems (2 g of fresh weight) were blended in 100 mL of 50 mM phosphate buffer for 1 min and filtered with a GF/A filter. This maximises enzyme dynamics detection by focusing on the area of our interest. The soluble fraction of the extract from each of the five biological replicates per retting point was analysed with five technical repetitions. All the enzyme activities were determined at 25°C and expressed by nmol/gMS/h, except polygalacturonase (expressed in μmol/gMS/h).

The peroxidase and phenol oxidase enzyme activities were measured by using 5 mM L‐DOPA (L‐3,4‐dihydroxyphenylalanine) as described previously (Sauvadet et al. 2016; Chabbert et al. 2020). Peroxidase enzyme hydrolyses hydrogen peroxide into water and molecular oxygen, and that reactive oxygen further oxidises L‐DOPA to D‐IQC (3‐hyroindole‐5,6‐quinone‐2‐carboxylate). The absorbance is taken at 460 nm to measure the enzyme activity.

Polygalacturonase activity was quantified by the DNS method and a galacturonic acid calibration curve, as reported by Bleuze et al. (2018). Enzyme endo‐polygalacturonase (EPG) breaks the α‐1,4‐glycosidic bonds in polygalacturonic acid and releases galacturonic acid monomer units with a free‐reducing sugar end group. They react with DNS and change the colour. The enzyme (800 μL) substrate (200 μL) mixture was added with DNS (500 μL) after 1 h of incubation and treated with a boiling water bath for 15 min to finish the reaction. The colour change of the reaction mixture is proportional to the free galacturonic monomers, that is, EPG activity. The absorbance change is measured at 540 nm to read the enzyme activity as reported by Chabbert et al. (2020).

Cellobiohydrolase, β‐D‐glucosidase, β‐D‐xylosidase, and β‐D‐galactosidase activities were measured by measuring the release of 4MUB (4‐methylumbelliferone) as described by Sauvadet et al. (2016) and Chabbert et al. (2020). 4MUB acts as a fluorophore. Esters of 4MUB do not fluoresce unless cleaved to release the fluorophore. The enzymatic hydrolysis of 4MUB‐containing substrate that is, MU‐β‐D‐glucopyranoside releases fluorescence which is proportional to the enzyme activity. The reaction mixture (in 96 deep well plates) was incubated for 3 h in the dark and measured at 460 nm using a microplate spectrophotometer CLARIOstar.

## 3 Results

### 3.1 Sequencing Quality

Assessing the adequacy of sequencing depth is crucial when studying microbial communities, and rarefaction curves are a valuable tool in this regard. Once the data is subsampled, filtered, and preprocessed (Tables S2–S5), rarefaction curves are constructed to identify the maximum diversity plateau for each sample (Figure S5).

For bacterial sequences, the number of raw reads averaged 47,980 ± 9270 for field stem samples (*n* = 16), 27,177 ± 2898 for field soil samples (*n* = 3), 42,964 ± 7773 for pilot unit stem samples (*n* = 5), and between 21,038 and 2646 for pilot unit soil samples (*n* = 2). These differences in sequencing depth reflect variations in sample type and microbial load, which may be influenced by retting conditions.

Fungal sequencing yielded 75,743 ± 22,289 reads for field stem samples (*n* = 16), 78,923 ± 9419 for field soil samples (*n* = 3), 81,504 ± 16,402 for pilot unit stem samples (*n* = 5), and between 93,742 and 95,743 for pilot unit soil samples (*n* = 2). The higher read counts observed in fungal sequences compared to bacterial sequences suggest that fungal communities may be more abundant during the retting process.

These results confirm that sequencing provides a comprehensive overview of microbial diversity during the retting process.

For the analysis of 18S rDNA sequencing, the percentage of reads from plants has been computed. To determine the proportions of plant and fungal material in each sample, the ratio between the number of raw reads and the number of reads obtained after the taxonomic assignment was calculated. The results indicate that plant DNA is poorly amplified for all samples (0.78% ± 0.95% and 0.41% ± 0.52% for field and pilot unit stem samples respectively, and 3.91% ± 1.58% for field soil samples and between 2.80% and 3.93% for pilot unit soil samples), except for the unretted stem sample (R0), for which the percentage of plant reads is high (26.91%) (Table S6).

### 3.2 Microbial Community Diversity

Alpha diversity of the microbial community is assessed by calculating species richness (Chao1 index), microbial community equilibrium (Shannon index), species evenness (Pielou index), and the Inverse Simpson index (Table S8). These indices enable us to identify the complexity and balance of microbial communities at different stages of retting.

The results reveal distinct patterns in microbial diversity between bacterial and fungal communities. It is observed that bacterial communities exhibit greater diversity than fungal communities for both field and pilot unit samples (including stems and soil).

Comparing the soil samples from the two retting conditions (in the field and the pilot unit), the alpha diversity indicators are similar, with a slightly higher diversity observed in the field retting condition (Table S7).

No significant difference is observed in bacterial community diversity and fungal community diversity between the different swaths 1, 3, and 4 of stem samples retted in the field (Table S9). In addition, according to the Venn diagram (Figure S6), numerous ASV (40% for the bacterial community and 59% for the fungal community) are mutual to the three studied swaths.

No significant difference in the dynamics of bacterial and fungal communities is observed for stem samples retted in the field (inverse Simpson index) (Table S9).

No significant difference (*p*‐value > 0.05) is observed in the bacterial and fungal community diversity of samples (stem vs. stem) between field retting and pilot unit retting (Table S10). Furthermore, the colonisation dynamics of bacterial and fungal communities are compared between the field and the pilot unit, and no significant difference is found, as indicated in Table S11.

About beta diversity, the non‐metric multidimensional scaling (NMDS) analysis based on Bray‐Curtis similarities (Figure S7) exhibits distinct groups within the microbial community structure of stem and soil samples (for both the field and the pilot unit). The stem samples are closely clustered on the right side of the graphs, while the soil samples are clustered together on the left side.

In return, the presence of two separate clusters in the NMDS plot could suggest some differences in the fungal community structure between the soil sampled from the field and the soil sampled from the pilot unit. It is important to approach these observations with caution, because of the limited number of soil samples and, in particular, the difficulty in performing statistical analyses on these results.

Principal Coordinate Analysis (PCoA) (membership analysis) based on Bray‐Curtis distances (Figure S9,10) confirms the NMDS analysis.

Similar to the NMDS analysis, the PCoA plot for fungal community membership of field and pilot unit soil samples also exhibits two distinct clusters.

The determination of the common ASVs fraction within both soils (in the field and the pilot unit) reveals these differences: 37.9% at the beginning of retting (R0) and 30.8% after 2 weeks of retting (R2) (Figure S8).

No important difference in the community membership of bacterial and fungal communities between field and pilot unit stem samples is observed. It can be noticed that the fungal communities of the stem samples (field and pilot unit) exhibit more similarity than the bacterial communities. This indicates that retting conditions may favour consistent microbial colonisation dynamics regardless of environmental differences.

**FIGURE 1:**
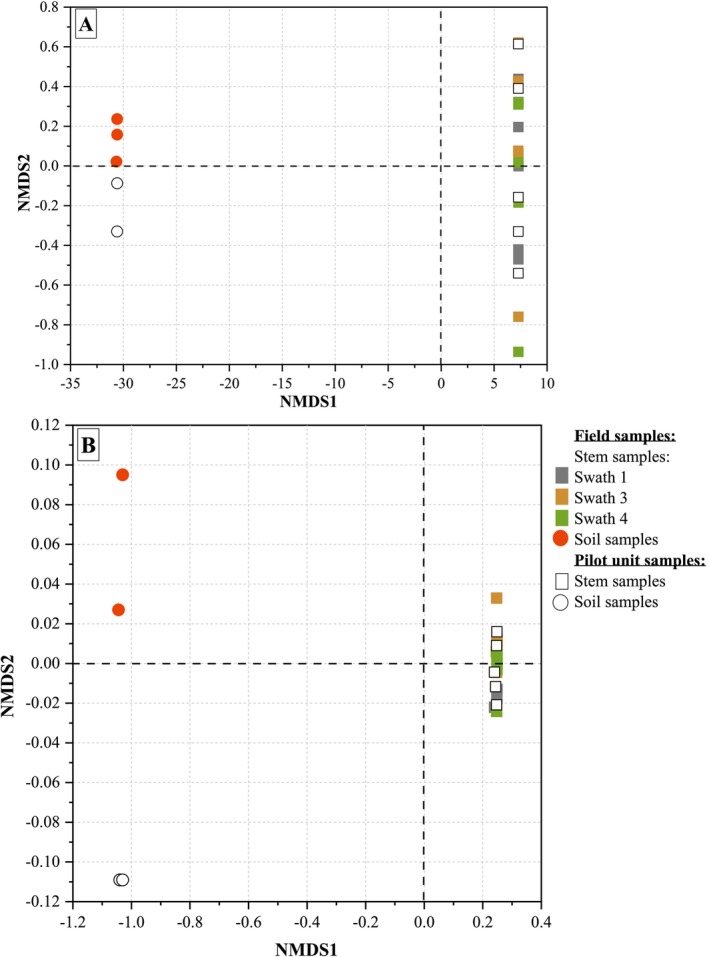
Non‐metric multidimensional scaling (NMDS) of bacterial (16S) (A) and fungal (18S) (B) community structure for field and pilot unit stem and soil samples.

The evolution of PCoA results of stem samples retted in the field and the pilot unit as a function of retting time is shown in Figure 1.

The bacterial community analysis (Figure 1A) reveals a similar trend in stem samples retted in the field (swaths 1, 3, and 4) and in the pilot unit. A convergence of bacterial community diversity from different swaths is observed after 3 weeks of retting (R3), indicating a clustering at R4 and R6 (circle in Figure 1A).

No significant difference in the dynamics of the bacterial communities colonising stem samples in the field (Tables S12 and S13) is noted.

In addition, the colonisation dynamics of bacterial communities between the field and the pilot unit stem samples exhibit no significant difference (Table S14).

A fungal divergence between swaths in terms of beta diversity is observed after 3 weeks of retting (R3). The fungal community analysis reveals a similar trend in stem samples retted in the field (swaths 1, 3, and 4) and in the pilot unit (Figure 1B).

However, no significant difference in the dynamics of the fungal communities colonising stem samples retted in the field (Table S13) is revealed.

The fungal community analysis reveals a similar trend in stem samples retted in the field (swaths 1, 3, and 4) and in the pilot unit.

Analysing the bacterial ASVs in common from the stem samples (in the field and the pilot unit) (Table S15), the percentage of common ASVs ranges from 46.4% to 57.1%, showing that nearly half or more of the bacterial species are shared between the swaths in the field and the one in the pilot unit, demonstrating an important overlap of the bacterial community of retted swaths in the field and the pilot unit.

For the fungal communities, a lower fungal diversity compared to bacterial diversity is observed. However, the percentage of common ASVs (ranging from 62.7% to 73.4%) is relatively high, also indicating an important overlap of the fungal community between stem samples. These results exhibit a significant degree of microbial community overlap between the two retting conditions for both bacterial and fungal populations, with a slight trend towards greater similarity in fungal communities.

### 3.3 Community Structure

#### 3.3.1 Bacterial Community Structure

**FIGURE 2:**
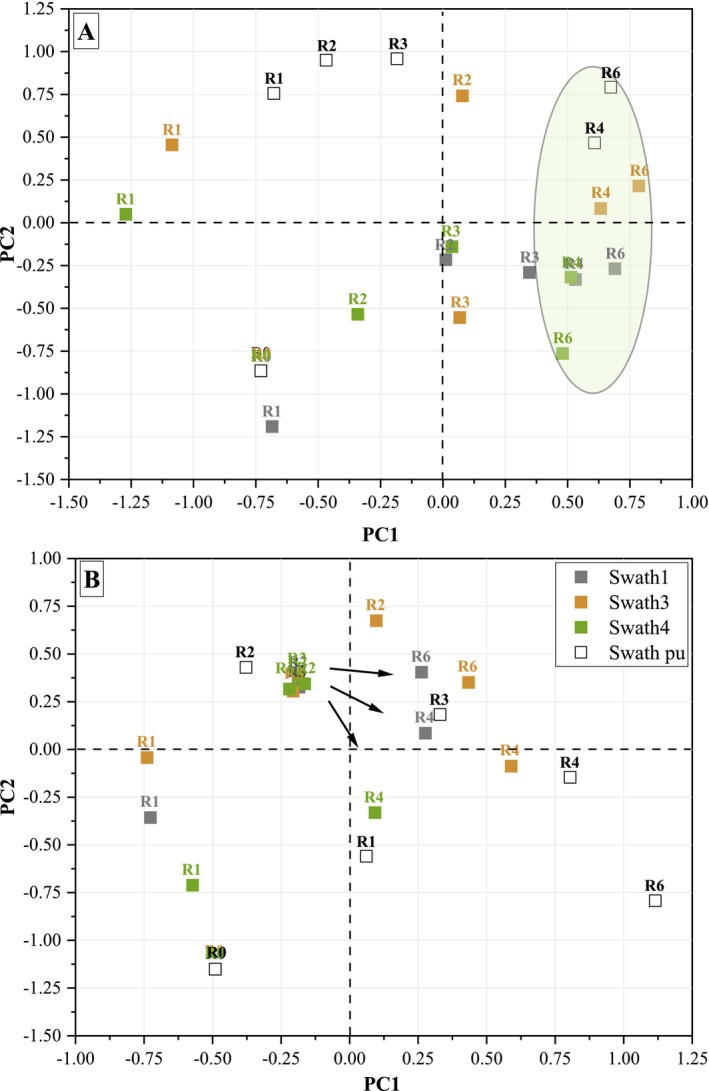
Principal coordinates analysis (PCoA) of bacterial (16S) (A) and fungal (18S) (B) community structure for the field (swaths 1, 3, and 4) and pilot unit (swath pu) stem samples during the different weeks of retting (R0‐R4, R6).

The bacterial community composition at the phyla level for both stem and soil samples from the field and pilot unit retting was analysed to reveal patterns of microbial succession during retting and their possible functional implications (Figure 2). It should be remembered that the relative abundances of phyla on the surface of the stems retted in the field were made by performing the average of the 3 swaths (1, 3, and 4). The relative abundances of bacterial phyla and classes in the three distinct swaths are also presented (Figure S10).

For hemp stems retted in the field, seven bacterial phyla are identified with four major phyla (relative abundance > 1%): *Proteobacteria*, *Bacteroidota*, *Actinobacteriota*, and *Myxococcota* with a large dominance of *Proteobacteria* during the 6 weeks of retting. The minor phyla (relative abundance < 1%) are *Firmicutes*, *Bdellovibrionota*, and *Acidobacteriota*. Both the unretted stems (R0) and the stems retted for 6 weeks (R6) exhibit analogous profiles regardless of the retting conditions. Nevertheless, a decrease of *Proteobacteria* relative abundance from 93.1% at R0 to 72.9% ± 10.9% at R6 and an increase in *Bacteroidota* relative abundance from 1.1% at R0 to 23.3% ± 11.5% at R6 are observed. This shift suggests a microbial turnover possibly linked to shifts in nutrient availability induced by plant tissue degradation. Such a shift may indicate the involvement of *Bacteroidota* in later stages of lignocellulosic material decomposition.

The phyla profiles obtained from stems retted within the pilot unit are similar to those mentioned above, with a large dominance of *Proteobacteria* and a great increase of relative abundance values of *Bacteroidota* at the end of the retting process. Although *Proteobacteria* has the highest prevalence during retting, the relative abundance values of *Proteobacteria* decrease to close to 60% at R6 while *Bacteroidota* increases to around 36% at R6. Moreover, *Actinobacteriota* relative abundance values remain stable around 1%–3% during retting, and a transient occurrence of *Firmicutes* (1.4%–2.8%) is observed during the retting process (R3).

Together, these results point to a reproducible community shift across retting environments and emphasise the central role of *Bacteroidota* in the decomposition process during the late stages of retting.

For soil samples, a greater variety of phyla compared to the stem samples is observed. This higher diversity reflects the complex microbial ecosystem in soil, which likely serves as a reservoir for microbial colonisation during retting. However, the changes in bacterial composition observed from the soil samples during retting are less pronounced than those in the stem samples.

For field soil samples, 10 phyla are identified, with *Proteobacteria*, *Bacteroidota*, *Actinobacteriota*, *Acidobacteriota*, *Myxococcota*, *Firmicutes*, and *Gemmatimonadota* as major phyla (with relative abundance values greater than 1%). During retting, similar relative abundance values are observed for the dominant phyla. *Proteobacteria, Bacteroidota, Actinobacteriota*, and *Firmicutes* exhibit slight variations from 46.0% (R0) to 55.5% after 4 weeks of retting (R4), 10.5% (R0) to 17.8% (R4), 29.1% (R0) to 16.5% (R4) and 2.8% (R0) to 1.4% (R4) respectively.

*Acidobacteriota*, *Myxococcota*, and *Gemmatimonadota* remain stable during retting at approximately 5%, 3%, and 2%, respectively. Relative abundance values of *Desulfobacterota‐B*, *Entotheonellaeota*, and *Nitrospirota* phyla are inferior to 1%. The relative abundances of phyla after 2 weeks of retting (R2) are comparable to those at 4 weeks of retting (R4).

For samples from the soil used for hemp culture (pilot unit), the profile observed at R0 is quite similar to the profile obtained from field soil samples with 10 major phyla, including *Proteobacteria*, *Bacteroidota*, *Actinobacteriota*, *Acidobacteriota*, *Myxococcota*, *Firmicutes*, *Gemmatimonadota*, and *Nitrospirota*, and two minor phyla, *Desulfobacterota‐B* and *Entotheonellaeota* (relative abundance < 1%). Similar relative abundance trends are observed between soil samples (at R0) and soil samples (at R2) regardless of the retting condition (field and pilot unit). Slight variations are then observed for *Proteobacteria, Actinobacteriota*, and *Firmicutes* from 42.9% (R0) to 57.7% at R2, 26.3% (R0) to 16.2% (R2) and 2.8% (R0) to 1.9% (R2) respectively. *Bacteroidota*, *Acidobacteriota*, *Myxococcota*, *Gemmatimonadota*, and *Nitrospirota* remain stable during retting at approximately 14%, 5%, 2%, 2%, and 1%, respectively, highlighting their persistent presence regardless of retting progression.

**FIGURE 3:**
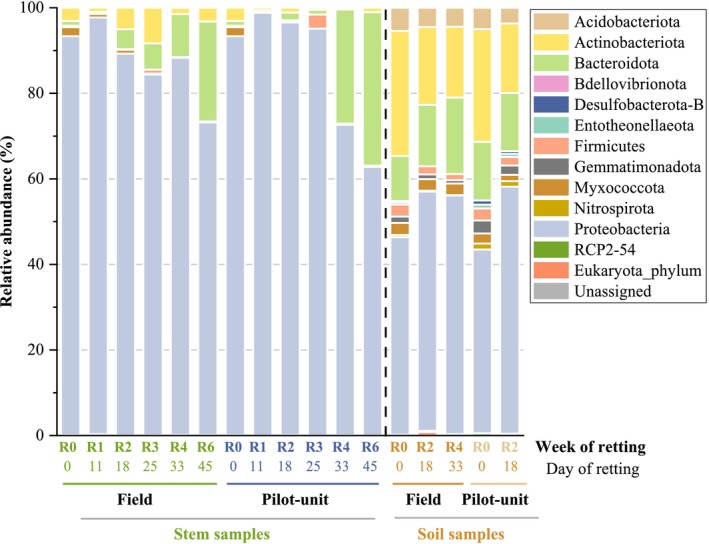
Bacterial relative abundance at phylum level in unretted and retted stem and soil samples in field and pilot unit over the retting period. R0: Unretted samples, R1, R2, R3, R4, and R6 correspond to retted samples after 1, 2, 3, 4, and 6 weeks of retting.

To examine the evolution in bacterial communities at a more specific level (genus level), a heatmap representing the top 10 bacterial ASV identified in stems from field and pilot unit samples is given in Figure 3.

The heatmap reveals that during retting, the most dominant bacterial genera are *Pantoae, Pseudomonas* (*Gammaproteobacteria* class), *Sphingomonas*, *Methylobacterium*, and *Rhizobium* (*Alphaproteobacteria* class). These genera displayed distinct temporal patterns likely linked to their ecological niches and functional roles in fibre degradation.

*Pseudomonas* and *Pantoea* genera are observed to increase at R1, then decrease at R2, and are observed in lower amounts until the end of retting (R6). On the other hand, *Sphingomonas* and *Rhizobium* genera increase at R2 and continue to increase until the end of retting (R6). The *Methylobacterium* genus has a high abundance at the beginning of retting (R0‐R1), then decreases at R2 and remains present at a constant rate until the end of retting (R6). These distinct temporal trajectories support the idea of functional succession during retting, where early colonisers (e.g., *Pseudomonas*, *Pantoea*) pave the way for later, more specialised taxa (e.g., *Sphingomonas*, *Rhizobium*), enabling efficient plant matter breakdown and microbiome stabilisation over time. This dynamic community restructuring appears consistent across both field and pilot unit conditions.

#### 3.3.2 Fungal Community Structure

**FIGURE 4:**
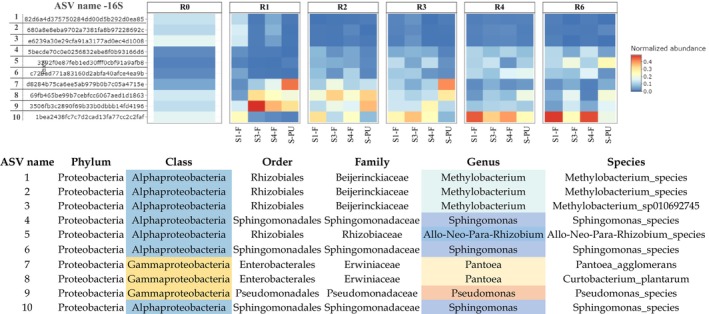
Heatmap of the relative abundance of the top 10 ASV of bacteria in stem field and pilot unit samples. S1‐F: Swath 1 retted in the field, S3‐F: Swath 3 retted in the field, S4‐F: Swath 4 retted in the field, and S‐PU: Swath retted in the pilot unit. ASV are arranged in ascending order of abundance, ranging from the least abundant (1) to the most abundant (10).

Figure 4 displays the fungal communities at the phylum level for stem and soil samples (field and pilot unit). As for bacterial phyla, the relative abundances of fungal phyla on the surface of the stems retted in the field were made by performing the average of the three swaths (1, 3, and 4). The relative abundances of fungal phyla and classes in the three distinct swaths are also performed (Figure S11). As for bacterial samples, an evolution in the fungal structure throughout retting is observed.

Two phyla are identified for hemp stem samples retted in the field and within the pilot unit: *Ascomycota* and *Basidiomycota*. *Ascomycota* dominates during the 6 weeks of retting, and its relative abundance increased from 51.0% at R0 to 80.0% ± 6% (field) and 89.4% (pilot unit) at R6. In contrast, *Basidiomycota* relative abundance values decrease during retting from 47.8% at R0 to 14.7% ± 2% (field) and 2.9% (pilot unit) at R6.

More fungal phyla are identified in the soil samples (field and pilot unit) than in the stem samples. Seven phyla are present in soil field samples with four major phyla (relative abundance > 1%): *Ascomycota*, *Basidiomycota*, *Mucoromycota* (formerly *Zygomycota*), and *Chytridiomycota*. Rare phyla, present only in soil samples at a low abundance (< 0.2%), are identified for the first time during the hemp retting process, such as *Blastocladiomycota*, *Zoopagomycota* (found in both soils from the field and the pilot unit), and *Olpidiomycota* (found only in the soil from the field).

A slight increase of *Ascomycota* during retting from 71.0% at R0 to 76.9% at R4 and a decrease of *Basidiomycota* relative abundance from 6.4% at R0 to 3.6% at R4 are observed. The relative abundance values of *Mucoromycota* and *Chytridiomycota* also decrease during retting from 4.7% and 3.0% at R0 to 2.4% and 0.8% at R4, respectively.

For pilot unit retting, the relative abundance values of *Ascomycota* vary from 61.9% at R0 to 58.8% at R2. A decrease in *Basidiomycota* relative abundance values from 2.6% at R0 to 1.6% at R2 is observed. The relative abundance values of *Mucoromycota* and *Chytridiomycota* also decrease from 9.0% and 1.1% at R0 to 3.4% and 0.8% at R2, respectively.

**FIGURE 5:**
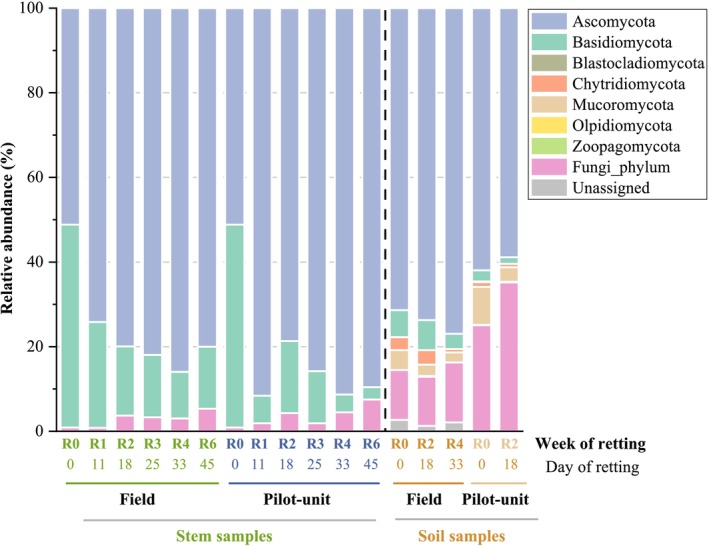
Fungal relative abundance at phylum level in unretted and retted stem and soil samples in field and pilot unit over the retting period. R0: Unretted samples, R1, R2, R3, R4, and R6 correspond to retted samples after 1, 2, 3, 4, and 6 weeks of retting.

A heatmap (Figure 5) represents the top 10 fungal ASV in stem field and pilot unit samples, indicating a higher diversity of fungal genera compared to bacterial genera (5 bacterial dominant genera). *Lectera*, *Plectosphaerellaceae_genus* (*Sordariomycetes* class), *Tilletiopsis* (*Exobasidiomycetes* class), *Didymosphaeria*, *Aureobasidium*, *Phoma*, and *Cladosporium* (*Dothideomycetes* class), *Vishniacozyma*, and *Filobasidiaceae_genus* (*Tremellomycetes* class) are identified as the most dominant fungal genera during retting. Throughout different retting weeks and under the two different retting conditions (field and pilot unit), the genus *Cladosporium* exhibits important dominance over all other fungal genera.

The findings seem to exhibit that the *Lectera* genus displays an increase in abundance towards the end of retting (R4–R6). *Plectosphaerellaceae genus*, *Didymosphaeria*, *Aureobasidium*, and *Phoma* exhibit a fluctuating trend during retting. The *Vishniacozyma* genus increases at R1, followed by a stable trend until the end of retting (R6). *Filobasidiaceae genus* decreases at R3 and then remains stable until the end of retting (R6). In contrast, the *Cladosporium* genus shows a notable increase at R1 and then remains stable until the end of retting (R6). On the other hand, *Tilletiopsis* decreases at R1 and continues to decline until the end of retting (R6).

After studying the structure and diversity of both retting bacterial and fungal communities, the functional potential will be assessed. These microbial communities play crucial roles during the retting process through the production of specific plant cell wall degrading enzymes. The prediction and measurement of the enzymatic activities over the retting period provide key insights into the active biological processes of retting.

### 3.4 Prediction of Potential Bacterial Enzymatic Activities

**FIGURE 6:**
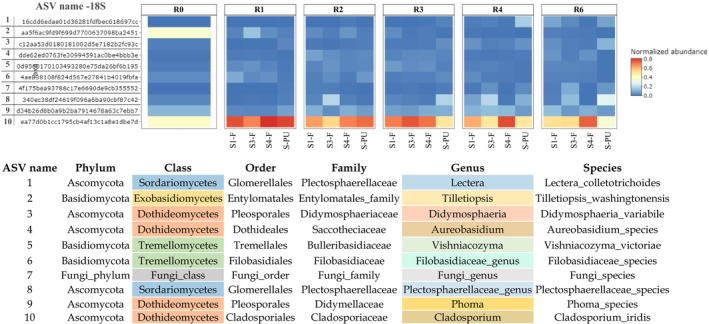
Heatmap of the relative abundance of the top 10 ASV of fungi in stem field and pilot unit samples. S1‐F: Swath 1 retted in the field, S3‐F: Swath 3 retted in the field, S4‐F: Swath 4 retted in the field, and S‐PU: Swath retted in the pilot unit. ASV are arranged in ascending order of abundance, ranging from the least abundant (1) to the most abundant (10).

The efficiency of hemp field retting is closely linked to microbial enzymatic degradation of plant cell wall polymers, particularly through hydrolytic enzymes targeting cellulose, hemicellulose, and pectin. The PICRUSt software was used to predict bacterial Carbohydrate Active Enzyme (CAZy) families (Djemiel et al. 2022) potentially involved in cell wall polymer degradation during field retting. Enzyme profiles were predicted for three retted swaths (swaths 1, 3, and 4), with the average enzyme distribution shown in Figure 6. Notably, the predicted enzymatic activities exhibited significant similarities across the swaths, as illustrated in the corresponding heatmaps presented in Figure S12, suggesting a functionally consistent bacterial community despite spatial variation.

These results provide indirect but valuable insight into the metabolic potential of the retting microbiome, particularly regarding its ability to drive lignocellulosic matter decomposition through coordinated enzymatic activity.

Figure 6 illustrates a diverse set of predicted enzymes (Table S15) involved in the degradation of plant cell wall polysaccharides, including cellulose, hemicelluloses, and pectins. In total, 14 CAZy families targeting pectins (Figure 6D), 12 targeting hemicelluloses (Figure 6C), and three targeting cellulose polymers are identified (Figure 6B).

During retting, the three hydrolytic enzyme potentials of cellulolytic enzymes exhibit distinct trends. The most important activity is observed for EC:3.2.1.21 (ß‐glucosidase enzyme), which displays an increasing activity from 1 to 6 weeks of retting. On the other hand, EC:3.2.1.4 (cellulase) increases after R2 and then decreases after R4 and until the end of retting. EC:3.2.1.91 (cellobiohydrolase) shows the lowest activity compared to other cellulolytic enzymes.

The hydrolytic enzyme potential of hemicellulolytic enzymes exhibits different levels of activity at different weeks of retting. The most important hemicellulolytic enzymatic activities are EC:3.2.1.23 (ß‐galactosidase) and EC:3.2.1.51 (α‐L‐fucosidase). Most enzyme activities, such as EC:3.2.1.23 (ß‐galactosidase), EC:3.2.1.177 (α‐D‐xyloside xylohydrolase), EC:3.2.1.25 (ß‐mannosidase), EC:3.2.1.139 (α‐glucuronidase), EC:3.2.1.55 (α‐L‐arabinofuranosidase), and EC:3.2.1.51 (α‐L‐fucosidase) decrease at R1 and then increase after 2 weeks of retting (R2), with the highest activity observed in the later stages (R4‐R6).

The hydrolytic enzyme potential of pectinolytic enzymes exhibits also different levels of activity at different weeks of retting. The most important pectinolytic activity during retting is observed for EC:3.2.1.23 (ß‐galactosidase). Enzyme activities such as EC:3.2.1.37 (xylan 1,4‐ß‐xylosidase), EC:3.2.1.55 (α‐L‐arabinofuranosidase), and EC:3.2.1.23 (ß‐galactosidase) show a decreasing activity at R1 and then a general trend of increasing activity after 2 weeks (R2) and until the end of retting. For the other pectinolytic enzymes, a fluctuating activity is observed during the different weeks of retting, possibly due to microbial competition, enzymatic redundancy, or stage‐specific substrate preferences.

### 3.5 Evolution of Enzyme Expression During Retting

**FIGURE 7:**
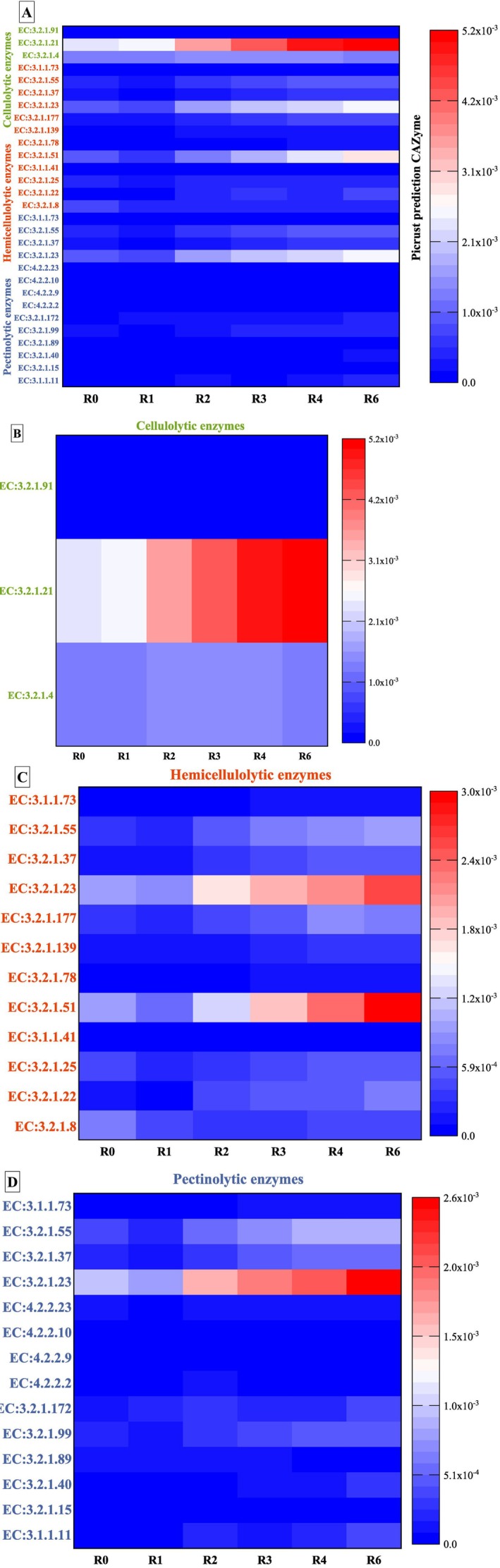
Heatmaps of predicted bacterial hydrolytic enzymes present in hemp field stem samples and generated by PICRUSt software. (A) Predicted cellulolytic, hemicellulolytic, and pectinolytic enzymes; (B) predicted cellulolytic enzymes; (C) predicted hemicellulolytic enzymes, and (D) predicted pectinolytic enzymes. Enzyme predictions are colour‐coded based on their raw abundance: The transition from blue through white to red reflects an increasing scale of enzyme occurrence from the lowest to the highest occurrence.

To complement the predictions made by the PICRUSt tool, potential hydrolytic (polygalacturonase, ß‐xylosidase, ß‐galactosidase, β‐D‐glucosidase, and cellobiose hydrolase) and oxidative (peroxidase, and phenoloxidase) enzymatic activities were directly measured during peeled outer stem tissue undergoing retting. The results of these enzymatic assays are presented in Figure 7.

Polygalacturonases, a key enzyme involved in pectin degradation, exhibit the highest activity during the early stage of retting (R1) followed by a progressive decline in activity in later stages (R6) (Figure 7A). This pattern aligns with the early depolymerization of middle lamellae pectin, facilitating the initial separation of plant fibres. In contrast, ß‐xylosidases and ß‐galactosidases, both of which contribute to the breakdown of hemicellulose, show increased activity after 4 weeks of retting. This suggests that hemicellulose becomes more accessible in the mid‐phase of fibre decomposition.

Cellulolytic enzymes, includingβ‐D‐glucosidases and cellobiose hydrolases, displayed the highest activity towards the final stages of the retting process (Figure 7B). These results support a triphasic model of retting: an initial phase characterised by pectin breakdown, a mid‐stage dominated by hemicellulose cleavage, and a late stage involving cellulose degradation, each phase associated with specific enzymatic activities and characteristic microbial actors.

Enzymes involved in degradation of lignin/phenolics, specifically peroxidases and phenoloxidases, exhibited their highest activity during the first week of retting (R1) (Figure 7C). This early oxidative activity may play a role in lignin breakdown, helping to open up the fibre structure and prepare it for the action of the hydrolytic enzyme.

Table S16 provides statistical validation of the enzyme dynamics during retting. Peroxidase activity is significantly higher at R0 compared to R1, R4, and R6, while phenoloxidase activity is significantly elevated in R0 and R1 relative to R4 and R6. However, no significant difference is observed in the activities of cellobiose hydrolase, ß‐xylosidase, ß‐glucosidase, and ß‐galactosidase across the different stages of retting. These results highlight the distinct temporal pattern of enzyme activity during retting. Peroxidase activity is highest at the initial stage of retting and decreases at later stages, while phenoloxidase activity decreases during the retting period. In contrast, the activity of the other enzymes does not vary significantly during retting. These results underline the importance of oxidative enzymes in the early phase of retting and suggest a more temporally sustained role for hydrolytic enzymes.

PICRUSt results reveal the presence of polygalacturonase (EC:3.2.1.15), ß‐xylosidase (EC:3.2.1.37), ß‐galactosidase (EC:3.2.1.23), ß‐glucosidase (EC:3.2.1.21) and cellobiose hydrolase (EC:3.2.1.91) enzymes. According to PICRUSt results, a low enzyme activity for polygalacturonase compared to the other pectinolytic enzymes is noted during retting. It displays a decreasing activity at R1, then an increase at R2, and finally a decreasing activity at R6. ß‐xylosidase, ß‐galactosidase, and ß‐glucosidase enzymes show a decreasing activity at R1, followed by an increased activity at R2 and until the end of retting. The same trend of evolution was observed for cellobiose hydrolase but shows a decreasing activity at R4.

## 4 Discussion

### 4.1 Retting Microbial Diversity

The evolution of the diversity of bacterial and fungal communities in the soil (field and pilot‐unit) during retting is similar for both the field and the pilot unit. Moreover, no difference was observed in the bacterial and fungal community diversity of stem samples between field retting and pilot unit retting. Thus, in the context of our work, this first result makes it possible to eliminate the effect of soil diversity on stem‐associated microbial assemblages, at least in terms of diversity indices.

**FIGURE 8:**
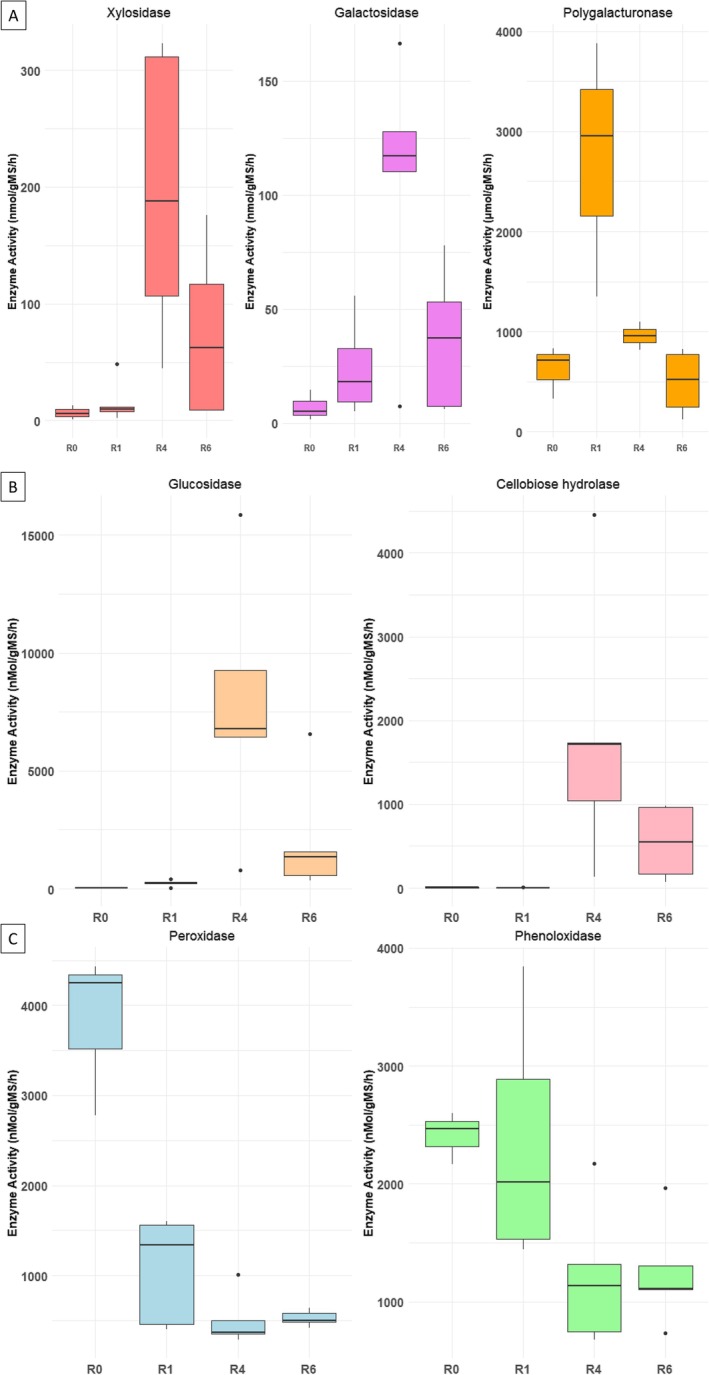
Enzymatic activities associated with hemp stems during field‐retting (A) polygalacturonase (μmol/gMS/h), ß‐xylosidase, and ß‐galactosidase (nMol/gMS/h); (B) cellobiose hydrolase and ß‐glucosidase (nMol/gMS/h); (C) phenoloxidase and peroxidase (nMol/gMS/h).

It has been reported that plants are the main factors determining the structure of soil microbial communities (Garbeva et al. 2006). Nowadays, the investigation of the relationships between plant diversity and soil microbial communities is increasing (Ke et al. 2015; Garbeva et al. 2006). In our work, common bacterial and fungal ASV were observed between soil and stem samples (Figure 8), pointing to a likely overlap and microbial exchange between these compartments.

During the retting process, it is probable that complex relationships between the microbial communities on the stems and those in the soil mediate the temporal dynamics of microbial diversity of stems. Hemp plants are part of the lignocellulosic biomass, which represents a complex ecosystem to be degraded by microorganisms. Thus, different microbial populations, particularly hemicellulolytic and cellulolytic ones (Ventorino et al. 2015), are present during the retting process, as it has been highlighted in our work. This implies that the decomposition of stems during retting leads to changes in microbial diversity within the soil. These changes result in the supply of organic substrates entering the soil, thereby promoting microbial diversity in the soil and interactions with plant microorganisms. Over two‐thirds of organic matter is refilled to the soil during hemp field retting (Adesina et al. 2020). Hence, it is highly probable that retting induces changes in the composition and structure of the stem and soil microbial community. Thus, retting appears to induce a bidirectional influence on microbial communities, simultaneously reshaping both stem and soil microbiota via substrate availability and microbial interactions.

The results of beta diversity analysis may suggest that the fungal communities in the soil samples from the field and the pilot unit may have some differences in the microbial composition. The observed low percentage of the common fungal ASVs fraction within both soils (in the field and the pilot unit) could be due to the taxonomic composition or differences in relative abundances of microbial groups. It can be suggested that differences in the physicochemical properties of the two different soil samples may impact the structure of the fungal communities. Considering the influence of abiotic and biotic factors on soil microbiomes (Ware et al. 2021), it is probable that these factors contribute to the observed differences in our study. The observed low percentage of the common fungal ASVs fraction within both soils (in the field and the pilot unit) could be due to the taxonomic composition or differences in relative abundances of microbial groups.

The analysis of bacterial and fungal communities in stem samples from the field and pilot unit reveals a high percentage of common ASVs for bacteria and fungi, demonstrating significant microbial community similarity between the two retting conditions. These findings align with the microbial diversity patterns observed in field‐grown hemp, where significant variations in bacterial and fungal community structures were detected across different compartments and fields (Ahmed et al. 2021). This emphasises the influence of environmental conditions, such as retting stages and hemp field diversity, on microbial community composition and dynamics (Ahmed et al. 2021; Dumigan and Deyholos 2024; Bou Orm et al. 2023).

### 4.2 Retting Microbial Structure

#### 4.2.1 Bacterial Community Structure

It should be noted that comparisons between the microbial communities identified in our study and those in other studies should be taken with caution due to the significant differences in ASV identification methods and taxonomic assignment databases employed.

Bacterial phyla such as *Proteobacteria*, *Bacteroidota*, *Actinobacteriota*, and *Firmicutes* identified in this study have been previously identified during retting of different fibrous plants (jute, flax, hemp, and bamboo) (Munshi and Chattoo 2008; Fu et al. 2011; Ribeiro et al. 2015; Zhao et al. 2016; Djemiel et al. 2017; Liu et al. 2017; Chabbert et al. 2020; Hasan et al. 2020; Law et al. 2020). Their presence across different fibre degradation environments supports the idea of a conserved core microbiome functionally adapted to lignocellulose decomposition. In addition, these phyla have been found during the degradation of different lignocellulosic biomass in natural conditions (Schellenberger et al. 2010; Ventorino et al. 2015). *Proteobacteria*, *Actinobacteriota*, and *Firmicutes* are known as hemicellulose degradation populations (Wang et al. 2016; Gavande et al. 2021) and have been identified as the major ones in lignin degradation within tropical forest soils (DeAngelis et al. 2011; Woo et al. 2014). *Proteobacteria* are specifically known for their ability to secrete specific pectin‐degrading enzymes (Ivanova et al. 2016; Liu et al. 2017), while it has been reported that the *Bacteroidota* phylum can degrade cellulose from plant material (Wang et al. 2016; Hernández et al. 2022).

These observations suggest that the bacterial community during retting is not only taxonomically conserved across systems but also functionally structured to match the sequential breakdown of plant polymers.

The decrease in the relative abundance of *Proteobacteria* in stem samples at the expense of an increase in *Bacteroidota* during retting (from R2 to R6) has been previously demonstrated during field retting of flax (Djemiel et al. 2017) and during hemp retting, which has been performed in a controlled environment (Law et al. 2020). In this context, the changes in the relative abundance of *Bacteroidota* versus *Proteobacteria* could serve as a bioindicator for retting progress. The transition from *Proteobacteria* to *Bacteroidota* dominance parallels the enzymatic shift from pectin degradation (early stage) to hemicellulose and cellulose breakdown (late stage), suggesting that taxonomic succession is functionally orchestrated (Bou Orm et al. 2024b).

Other phyla that have been observed only within soil samples such as *Acidobacteriota, Gemmatimonadota*, and *Nitrospirota* have also recently been observed during flax and hemp retting in soil and stem samples (Djemiel et al. 2017; Law et al. 2020). *Acidobacteria* is known for its ability to degrade various plant compounds such as cellulose, hemicellulose, pectin, and lignin, indicating its potential importance in plant biomass degradation (DeAngelis et al. 2011; Štursová et al. 2012; Lacerda et al. 2017; López‐Mondéjar et al. 2022). *Gemmatimonadota* is considered crucial for deconstructing biomass and can break down hemicellulose and cellulose (D'haeseleer et al. 2013), as well as playing a role in lignin deconstruction (D'haeseleer et al. 2013; Li et al. 2022; Tom et al. 2022). Similarly, *Nitrospirota* is also involved in lignin degradation (Li et al. 2022). These results highlight the potential for previously unknown bacterial communities to play an essential role in the degradation of plant biomass and the importance of exploring the microbial diversity present in the soil during the retting process.

Rare bacterial phyla such as *Bdellovibrionota*, *Desulfobacterota*, *Entotheonellaeota*, and *Myxococcota* are identified for the first time during the retting process.

A recent investigation of the microbial communities in cellulolytic consortia isolated from lignocellulosic substrates reveals the presence of both phyla *Bdellovibrionota* and *Myxococcota* (Wang et al. 2021a; Gladkov et al. 2022). In addition, another study exhibits a positive correlation between *Bdellovibrionota* and *Myxococcota* and the β‐glucosidase enzyme, which is involved in cellulose degradation (Ma et al. 2022). The consortium *Desulfobacterota* and *Myxococcota* has been identified to play a role in lignin degradation (Li et al. 2022). Furthermore, *Myxococcota* can degrade hemicellulose by secreting glycosyl hydrolase enzyme (Murphy et al. 2021; Gladkov et al. 2022). The *Entotheonellaeota* phylum has been detected during composting processes that involve the decomposition of organic matter, including lipids, cellulose, and lignin (Araujo et al. 2021). Although these phyla may not be considered as retting agents, previous studies highlight that rare taxa can play a significant role in ecosystem functions, even if their contributions are less understood and more difficult to reveal than those of more abundant taxa (Logares et al. 2013; Shade et al. 2014; Kurm et al. 2019). In our study, the identification of rare phyla during the retting process highlights the importance of deepening the roles of these less well‐understood taxa in the retting ecosystem process. In addition, the presence of phyla known for their ability to degrade cellulose, hemicellulose, pectin, and lignin further emphasises the significance of bacterial communities in retting.

Concerning the bacterial genera identified during retting, the *Pantoea* genus has previously been identified during the retting of flax and hemp (Ribeiro et al. 2015; Djemiel et al. 2017). It has been reported to possess a significant capacity for lignocellulose degradation (Ma et al. 2018). It has been demonstrated that the *Pantoea* genus contains genes that encode carbohydrate‐active enzymes (CAZymes), which are primarily active on hemicellulose and simpler sugars (Adams et al. 2011). *Pantoea* species are also capable of producing pectinase (Feng et al. 2016), polygalacturonase (Rafique et al. 2016), and pectin/pectate lyase (Khalaf and Raizada 2020), thereby facilitating pectin degradation. Furthermore, they exhibit lignin degradation activities (Tao et al. 2022).

*Pseudomonas* genus has been previously associated with flax and hemp retting (Ribeiro et al. 2015; Djemiel et al. 2017; Liu et al. 2017; Law et al. 2020). *Pseudomonas* species are known to secrete enzymes that degrade plant cell walls, such as pectinases, cellulases, and proteases (Munshi and Chattoo 2008; Tao et al. 2022). Due to their pectinolytic and hemicellulolytic activities, *Pseudomonas* species are considered important natural retting agents for hemp stems (Liu et al. 2017; Law et al. 2020; Balthazar et al. 2022; Tao et al. 2022; Zhang et al. 2023).

The *Sphingomonas* genus has previously been detected during the retting process of flax, hemp, and bamboo (Fu et al. 2011; Ribeiro et al. 2015; Djemiel et al. 2017). This genus is recognised as one of the hemicellulolytic genera and can break down lignocellulosic material (Pandit et al. 2016; Tao et al. 2022).

The *Rhizobium* genus has been linked to flax and hemp retting in prior research (Ribeiro et al. 2015; Djemiel et al. 2017; Liu et al. 2017) and is one of the bacteria able to degrade lignocellulose (Zainudin et al. 2013; Tao et al. 2022). The dominance of taxa such as *Sphingomonas* and *Rhizobium* during periods of increased hemicellulolytic enzyme activity further supports their potential functional role in the retting process. These genera may serve as bioindicators for optimal retting stage assessment.

*Methylobacterium* is recognised as a methylotrophic bacterium for its ability to use methanol, which is produced during pectin degradation, as a source of carbon and energy (Renier et al. 2011; Zhu et al. 2016). This genus has been identified as a cellulolytic strain (Hu et al. 2014) and is also known for its ability to degrade lignin (Hernández et al. 2022).

#### 4.2.2 Fungal Community Structure

Four phyla among seven fungal phyla: *Ascomycota*, *Basidiomycota*, *Chytridiomycota*, and *Mucoromycota* present in stem and soil samples are associated with hemp and flax retting (Ribeiro et al. 2015; Djemiel et al. 2017; Liu et al. 2017).

*Ascomycota* fungi are known for their ability to secrete various carbohydrate‐active enzymes (CAZyme) that are involved in the degradation of plant biomass (cellulose, hemicellulose, lignin, and pectin), making them active microorganisms during retting (Challacombe et al. 2019; Song et al. 2019; Dong et al. 2021). The pectinolytic activities of *Ascomycota* species are particularly important for improving fibre decohesion during retting (Battaglia et al. 2011; Gacura et al. 2016; Challacombe et al. 2019). On the other hand, the *Basidiomycota* phylum is highly effective in degrading lignocellulosic material and has a high potential for cellulose degradation (Tian et al. 2010; Battaglia et al. 2011; Rytioja et al. 2014; Eichlerová et al. 2015; Perkins et al. 2021).

The progressive replacement of the *Ascomycota* phylum by *Basidiomycota* during retting and plant decomposition processes has been largely highlighted by different authors (Lindahl and Boberg 2008; Voriskova and Baldrian 2013; Purahong et al. 2016; Djemiel et al. 2017). During retting, the challenge is to avoid the fibres crystalline cellulose being damaged as the mechanical properties of fibres could be greatly reduced (Mazian et al. 2018; Bou Orm et al. 2024b). Therefore, analysing the fungal community and the changes in the relative abundance of *Ascomycota* and *Basidiomycota* could serve as a secondary bioindicator of retting evolution.

The *Mucoromycota* fungi are primarily associated with plants and saprotrophic species (Reynolds et al. 2023). While they are not actively involved in the degradation of lignocellulosic materials, as they have limited cellulase production (Dzurendova et al. 2022), they do produce pectinolytic (Alves et al. 2002; Dzurendova et al. 2022) and ligninolytic enzymes (Perkins et al. 2021; Reynolds et al. 2023). In contrast, *Chytridiomycota* can decompose lignocellulose materials (Song et al. 2019; Reynolds et al. 2023).

About rare phyla, *Blastocladiomycota* (0.02% in field and 0.16% in pilot‐unit) has the potential to produce a limited number of carbohydrate‐active enzymes (CAZymes) and cellulolytic enzymes (Sista Kameshwar and Qin 2019). *Zoopagomycota* (0.13% in field and pilot‐unit) which are known as parasites of fungi, could also produce a limited number of CAZymes such as ligninolytic and cellulolytic enzymes (Sista Kameshwar and Qin 2019). On the other hand, *Olpidiomycota* (0.05% in field) are capable of decomposing plant residuals (Zhang et al. 2019).

Concerning the fungal genera identified during retting, only *Aureobasidium*, *Phoma*, and *Cladosporium* genera have been previously identified during flax and hemp retting (Sharma 1986; Koivula et al. 2004; Ribeiro et al. 2015), for contributing to lignocellulose degradation (Senthilguru et al. 2011; Bredon et al. 2018; Ma et al. 2018). *Lectera* and *Plectosphaerellaceae* genera are typically plant pathogens and soil fungal communities (Giraldo and Crous 2019; Liu et al. 2019; Wang et al. 2021b). The *Tilletiopsis* genus can produce exo‐ and endo‐β‐1,3‐glucanase (Urquhart and Punja 2002). Some species are dominant during agricultural waste composting and enable the production of microbial fat from lignocellulose (Yu et al. 2015; Li et al. 2011). The *Didymosphaeria* genus is involved in lignin degradation (Arredondo‐Santoyo et al. 2020). The *Vishniacozyma* genus is involved in lignocellulose degradation (Zhou et al. 2022). Some *Filobasidiaceae species* can generate pectinases (Merín et al. 2014). Several *Cladosporium* species are known to produce various lignocellulolytic enzymes, including cellulases (Mushimiyimana and Tallapragada 2013), ligninases, and xylanases (Ji et al. 2014). Overall, these results provide deep insights for the understanding of actively lignocellulose‐degrading fungi in the retting process.

#### 4.2.3 Towards Biomarker Identification

The retting process is influenced by various factors, including plant species, geographical locations, and climate conditions, affecting the microbial communities present during retting (Réquilé et al. 2021; Angulu and Gusovius 2024). However, our study highlights common bacterial (*Proteobacteria*, *Actinobacteriota*, *Bacteroidota*, and *Firmicutes*) and fungal (*Ascomycota* and *Basidiomycota*) taxa present during retting, regardless of the plant species or soil characteristics.

*Proteobacteria*'s dominance during early retting stages underscores their role as primary decomposers, secreting pectin‐degrading enzymes such as polygalacturonase. The subsequent increase in *Bacteroidota* relative abundance reflects a shift towards hemicellulose and cellulose degradation, highlighting their importance in later stages of retting. Similarly, fungal communities were dominated by *Ascomycota* throughout retting, indicating their specialisation in degrading lignocellulosic substrates.

The dynamics of microbial communities during the retting process can serve as bioindicators for tracking the progression of retting. Monitoring the relative abundance of bacterial (*Bacteroidota* vs. *Proteobacteria*) and fungal (*Ascomycota* vs. *Basidiomycota*) phyla changes suggests two potential bioindicators of the retting process.

To summarise, by gaining a better understanding of how these microorganisms evolve during retting, the process can be optimised and controlled. In addition, the similarities between the retting microbiome and microbial communities involved in natural plant decomposition suggest the significance of further exploration and understanding of the microbial communities involved in retting.

### 4.3 Retting Predicted Enzymatic Activities

While the PICRUST software can provide valuable insights into the potential functional capabilities of bacterial communities based on their 16S rRNA gene profiles, different limitations to using this approach should be noted. These limitations include (i) the completeness and quality of the reference genome database, (ii) the assumption that the genetic potential of a microbial community can be inferred solely from its taxonomic composition, and (iii) the potential mismatch between the predicted enzymatic potential and the actual enzymatic activity (Langille et al. 2013; Djemiel et al. 2017; Douglas et al. 2019). It is important to note that the presence of a taxon containing a gene encoding a specific enzyme in its genome does not necessarily imply that: (i) the taxon is alive (PCR amplification can also occur in dead organisms), (ii) it expresses the gene and produces the protein, (iii) the enzyme is active, (iv) the substrate of the enzyme is available. Overall, the accuracy and reliability of PICRUSt predictions should be carefully evaluated, especially when dealing with complex microbial communities (as for retting) or environments with low microbial diversity (Langille et al. 2013; Louca et al. 2018). Future studies should validate these predictions using functional assays to identify active microbial populations and their corresponding enzymes. In addition, exploring abiotic factors such as temperature and moisture could provide a more comprehensive understanding of how environmental conditions shape microbial dynamics during retting.

In our work, we observed a clear succession of potential enzymatic activities that correspond to shifts in the bacterial community during retting. Specifically, the dynamic profile of hydrolytic enzyme activities during retting suggests that different bacterial taxa contribute to distinct phases of fibre degradation. Thus, these observations highlight the dynamic nature of the retting process and may help in understanding and improving retting.

Fungi are important producers of extracellular hydrolytic enzymes that can break down various components of cell wall polymers, including cellulose, hemicellulose, and pectin (Aro et al. 2005; Djemiel et al. 2017). Studying the enzymatic activities of fungi would be an important addition to bacterial retting analysis.

### 4.4 Retting Enzymatic Activities

The analysis of retting enzyme activity indicates that polygalacturonases exhibit peak activity during the early retting stages, followed by a decline in activity in later stages. This could indicate that pectin degradation is an early step in the retting process. This aligns with the predominance of pectinolytic bacteria such as *Pseudomonas* in early retting. In contrast, ß‐xylosidases and ß‐galactosidases show increased activity after 4 weeks, suggesting that hemicellulose breakdown is slower than pectin degradation during retting, corresponding with the rise of bacterial taxa capable of hemicellulose hydrolysis, such as *Sphingomonas*. Cellulolytic enzyme activity peaks at the later retting stages, reinforcing cellulose degradation as the final step, coinciding with the emergence of cellulolytic bacteria such as *Methylobacterium*. This sequential enzymatic progression suggests a structured microbial succession driven by anatomical organisation of polysaccharides in the stem and fibre chemical complexity. These findings reinforce the potential to manage retting duration and quality through monitoring of key microbial and enzymatic indicators. In addition, these findings align with previous studies on flax dew retting (Chabbert et al. 2020; Mukherjee et al. 2024), and hemp dew retting (Liu et al. 2017; Bleuze et al. 2018), which also report a stepwise increase in enzymatic activity over time. Among cell wall components, cellulose exhibits the highest resistance to degradation due to its crystalline structure (Lynd et al. 2002), while pectin and hemicellulose degrade more readily due to amorphous nature and lower degree of polymerisation (Gilbert 2010). Lignin, with its complex cross‐linked structure, presents additional degradation challenges (Ralph et al. 2004).

Taken together, these results support a temporally regulated enzyme‐mediated retting process, where distinct enzymatic activities target specific plant cell wall components at different stages.

Metagenomic analysis confirms the presence of a diverse microbial community with enzymatic potential, aligning with observed enzyme activity trends and providing insight into microbiome– regulated retting dynamics.

While microbial enzymes predominantly drive retting, early‐stage enzymatic activity may also involve plant‐derived enzymes (Bleuze et al. 2018; Chabbert et al. 2020). Some plants have been found to exhibit continued metabolic activity even after harvest, which is often attributed to the presence of biochemically active plant enzymes (Moser 1995). Factors such as plant species, temperature, and humidity influence enzyme longevity, with endogenous plant enzymes, such as proteases and peroxidases, potentially contributing to early tissue degradation (Foyer and Noctor 2005; Li et al. 2019). Recent metaproteomic studies on flax dew retting further reveal that enzymes like galactosidases (GH35) and pectin esterases (ce8, ce13) remain active throughout retting (Mukherjee et al. 2024). This underscores the need for cautious interpretation of overall enzyme activity measurements.

It should be noted that both potential enzyme dynamics and measured enzyme activities display some similar trends with a time shift between the predicted and the measured enzyme activities. This temporal shift may reflect differences between genetic potential and actual microbial activity, highlighting the need for integrative approaches that combine functional advanced meta‐omics techniques (metaproteomics, meta‐transcriptomics, and meta‐metabolomics) with enzyme assays.

## 5 Conclusion

Considering the increasing demand for environmentally friendly fibres, it is becoming imperative to develop sustainable production systems that meet evolving environmental regulations. Therefore, understanding the retting process is crucial, as it significantly influences fibre quality and properties.

The convergence across swaths and retting stage, combined with relatively stable diversity metrics, suggests that retting drives a deterministic microbial succession. This succession aligns with the biochemical structure of the hemp cell wall and the sequential enzymatic degradation observed, supporting a structured, phase‐specific microbial colonisation. The bacterial and fungal communities appear to transition from pectin‐degraders in early stages to more specialised cellulolytic taxa, reflecting the structural composition of hemp tissues at each retting phase.

These results suggest specific bacterial and fungal taxa could serve as bioindicators to monitor retting progression.

Future research should explore metagenomics and metaproteomics to validate microbial functions, identify unknown enzymes, and gain a deeper understanding of retting‐associated interactions. A multimodal approach combining chemical, biochemical, molecular biology, and physicochemical methods can provide valuable insights about the different factors contributing to the changes occurring during field retting. This could potentially lead to a better understanding and more efficient management of the retting process, ultimately reducing the variability of hemp fibres.

## Author Contributions

**Eliane Bou Orm:** methodology, validation, visualization, writing – review and editing, formal analysis, data curation, conceptualization, investigation, writing – original draft, project administration. **Suvajit Mukherjee:** investigation, data curation, writing – review and editing. **Etienne Rifa:** software, data curation, writing – review and editing. **Anne Créach:** resources, supervision, writing – review and editing. **Sébastien Grec:** resources, supervision, writing – review and editing. **Sandrine Bayle:** resources, supervision, funding acquisition, project administration, conceptualization, methodology, writing – review and editing. **Jean‐Charles Benezet:** resources, supervision, funding acquisition, project administration, conceptualization, methodology, writing – review and editing. **Anne Bergeret:** resources, supervision, funding acquisition, project administration, conceptualization, methodology, validation, writing – review and editing, investigation. **Luc Malhautier:** resources, supervision, funding acquisition, project administration, conceptualization, methodology, validation, investigation, writing – review and editing.

## Conflicts of Interest

The authors declare no conflicts of interest.

## Supporting information


**Figure S1.** (A) Sampling sites: Drôme Chanvre, Mirabel et Blacons, France (green icon), laboratory site, Alès, France (grey icon), and Mas de la Valus, Bouquet, France (red icon). (B) The orange circle indicates the location of the retting site relative to the country map (France). The maps are generated on OpenStreetMap.
**Figure S2.** Experimental site during the field retting campaign. The numbers correspond to the distance (in meters) between swaths.
**Figure S3.** Photograph of the pilot unit illustrating the laboratory‐scale retting process. The pilot unit is filled with gravel/pozzolan (10 cm high) (1) covered by a layer of geotextile (2), 15 cm of soil (3), and the hemp stems (4).
**Figure S4.** Sampling plan used during the retting campaign in the field and in the pilot unit.
**Figure S5.** Rarefaction curves corresponding to bacterial (16S) and fungal (18S) domains for all samples.
**Figure S6.** Venn Diagram illustrating the shared bacterial (A) and fungal (B) ASV among swaths 1, 3, and 4 retted in the field.
**Figure S7.**Venn diagram of all common bacterial (A) and fungal (B) ASV between soil and stem samples (R0 and R4) belonging to the swath 4 retted in the field.
**Figure S8.** Venn diagram of all common bacterial (A) and fungal (B) ASV between soil field and soil of the pilot unit samples (R0 and R2).
**Figure S9.** Principal coordinates analysis (PCoA) of bacterial (16S) (A) and fungal (18S) (B) community structure for field and pilot unit stem and soil samples.
**Figure S10.** Bacterial relative abundance at phylum (A) and class (B) levels in unretted and retted stem samples (swaths 1, 3, and 4) in the field over time. R0: unretted samples, R1, R2, R3, R4, and R6 correspond to retted samples after 1, 2, 3, 4, and 6 weeks of retting.
**Figure S11.** Fungal relative abundance at phylum (A) and class (B) levels in unretted and retted stem samples (swaths 1, 3, and 4) in the field over time. R0: unretted samples, R1, R2, R3, R4, and R6 correspond to retted samples after 1, 2, 3, 4, and 6 weeks of retting.
**Figure S12.** Heatmaps of predicted bacterial hydrolytic enzymes present in each swath of hemp field stem samples (swaths 1, 3, and 4) and generated by PICRUSt software. (A) predicted cellulolytic enzymes; (B) predicted hemicellulolytic enzymes, and (C) predicted pectinolytic enzymes. Enzyme predictions are colour‐coded based on their raw abundance: the transition from blue through white to red reflects an increasing scale of enzyme occurrence from the lowest to the highest occurrence.

**Table S1.** qPCR reaction conditions for targeting bacterial and fungal communities.
**Table S2.** PCR1 Primers with Half Illumina Adapters for 16S and 18S sequencing.
**Table S3.** Table showing the total sum of bacterial reads (16S) for each stem and soil sample (field retting) at each step of the bioinformatics processing. The last numbers in the “reads tracking” table give the total raw sum of ASVs present in each sample.
**Table S4.** Table showing the total sum of bacterial reads (16S) for each stem and soil samples (pilot unit) at each step of the bioinformatics processing.
**Table S5.** Table showing the total sum of fungal reads (18S) for each stem and soil sample (field retting) at each step of the bioinformatics processing.
**Table S6.** Table showing the total sum of fungal reads (18S) for each stem and soil sample (pilot‐unit) at each step of the bioinformatics processing.
**Table S7.** Proportion of fungal and plant material for each sample.
**Table S8.** Bacterial and fungal alpha‐diversity of soil and stem samples (field and pilot unit retting).
**Table S9.** Bacterial and fungal alpha‐diversity (Shannon and invsimpson indices) of stem samples during the different weeks of field retting by TukeyHSD test. *, *p* < 0.05; **, *p* < 0.01; ***, *p* < 0.001.
**Table S10.** Bacterial and fungal alpha‐diversity (Shannon and Invsimpson indices) of stem samples retted in the field and in the pilot unit by TukeyHSD test. *, *p* < 0.05; **, *p* < 0.01; ***, *p* < 0.001.
**Table S11.** Comparisons of bacterial and fungal alpha‐diversity (Shannon and Invsimpson indices) of stem samples between the different weeks of retting in the field and in the pilot‐unit by TukeyHSD test. *, *p* < 0.05; **, *p* < 0.01; ***, *p* < 0.001.
**Table S12.** Bacterial and fungal beta‐diversity of stem samples retted in the field and in the pilot‐unit by permanova Adonis test. *, *p* < 0.05; **, *p* < 0.01; ***, *p* < 0.001.
**Table S13.** Bacterial and fungal beta‐diversity of stem samples during the different weeks of field retting by permanova Adonis test. *, *p* < 0.05; **, *p* < 0.01; ***, *p* < 0.001.
**Table S14.** Comparisons of bacterial and fungal beta‐diversity of stem samples between the different weeks of retting in the field and in the pilot‐unit by TukeyHSD test. *, *p* < 0.05; **, *p* < 0.01; ***, *p* < 0.001.
**Table S15.** Comparison of common and total amplicon sequence variants (ASV) in stem samples retted in the field (swaths 1, 3 and 4) and in the pilot unit for bacterial (16S) and fungal (18S) community.
**Table S16.** Enzyme PICRUSt nomenclature.
**Table S17.** Enzymatic activities of stem samples retted in the field by *T*‐test pairwise. *, *p* < 0.05; **, *p* < 0.01; ***, *p* < 0.001.

## Data Availability

The data that supports the findings of this study are available in the Supporting Information of this article.

## References

[emi470102-bib-0001] Adams, A. S. , M. S. Jordan , S. M. Adams , et al. 2011. “Cellulose‐Degrading Bacteria Associated With the Invasive Woodwasp Sirex Noctilio.” ISME Journal 5: 1323–1331. 10.1038/ismej.2011.14. 21368904 PMC3146269

[emi470102-bib-0002] Adesina, I. , A. Bhowmik , H. Sharma , and A. Shahbazi . 2020. “A Review on the Current State of Knowledge of Growing Conditions, Agronomic Soil Health Practices and Utilities of Hemp in the United States.” Agriculture 10: 40129. 10.3390/agriculture10040129.

[emi470102-bib-0003] Ahmed, B. , L. B. Smart , and M. Hijri . 2021. “Microbiome of Field Grown Hemp Reveals Potential Microbial Interactions With Root and Rhizosphere Soil.” Frontiers in Microbiology 12: 741597. 10.3389/fmicb.2021.741597. 34867858 PMC8634612

[emi470102-bib-0004] Alves, M. H. , G. M. Campos‐Takaki , A. L. Figueiredo Porto , and A. I. Milanez . 2002. “Screening of Mucor spp. For the Production of Amylase, Lipase, Polygalacturonase and Protease.” Brazilian Journal of Microbiology 33: 325–330. 10.1590/S1517-83822002000400009.

[emi470102-bib-0005] Amaducci, S. , D. Scordia , F. H. Liu , et al. 2015. “Key Cultivation Techniques for Hemp in Europe and China.” Industrial Crops and Products 68: 2–16. 10.1016/j.indcrop.2014.06.041.

[emi470102-bib-0006] Angulu, M. , and H.‐J. Gusovius . 2024. “Retting of Bast Fiber Crops Like Hemp and Flax—A Review for Classification of Procedures.” Fibers 12, no. 3: 28. 10.3390/fib12030028.

[emi470102-bib-0007] Araujo, A. S. F. , A. P. d. A. de Pereira , J. E. L. Antunes , et al. 2021. “Dynamics of Bacterial and Archaeal Communities Along the Composting of Tannery Sludge.” Environmental Science and Pollution Research 28, no. 45: 64295–64306. 10.1007/s11356-021-15585-9. 34304356

[emi470102-bib-0008] Aro, N. , T. Pakula , and M. Penttilä . 2005. “Transcriptional Regulation of Plant Cell Wall Degradation by Filamentous Fungi.” FEMS Microbiology Reviews 29: 719–739. 10.1016/j.femsre.2004.11.006. 16102600

[emi470102-bib-0009] Arredondo‐Santoyo, M. , J. Herrera‐Camacho , M. S. Vázquez‐Garcidueñas , and G. Vázquez‐Marrufo . 2020. “Corn Stover Induces Extracellular Laccase Activity in *Didymosphaeria sp*. (Syn. = *Paraconiothyrium sp*.) and Exhibits Increased In Vitro Ruminal Digestibility When Treated With This Fungal Species.” Folia Microbiologica (Praha) 65: 849–861. 10.1007/s12223-020-00795-4. 32372279

[emi470102-bib-0010] Balthazar, C. , D. L. Joly , and M. Filion . 2022. “Exploiting Beneficial *Pseudomonas spp*. for *Cannabis* Production.” Frontiers in Microbiology 12: 1–21. 10.3389/fmicb.2021.833172. PMC8795690 35095829

[emi470102-bib-0011] Battaglia, E. , I. Benoit , J. van den Brink , et al. 2011. “Carbohydrate‐Active Enzymes From the *Zygomycete* Fungus *Rhizopus Oryzae*: A Highly Specialized Approach to Carbohydrate Degradation Depicted at Genome Level.” BMC Genomics 12, no. 1: 38. 10.1186/1471-2164-12-38. 21241472 PMC3032700

[emi470102-bib-0012] Beauvais, F. , O. Cantat , P. Le Gouée , et al. 2022. “Consequences of Climate Change on Flax Fiber in Normandy by 2100: Prospective Bioclimatic Simulation Based on Data From the ALADIN‐Climate and WRF Regional Models.” Theoretical and Applied Climatology 148: 415–426. 10.1007/s00704-022-03938-4.

[emi470102-bib-0013] Bell, C. W. , B. E. Fricks , J. D. Rocca , J. M. Steinweg , S. K. McMahon , and M. D. Wallenstein . 2013. “High‐Throughput Fluorometric Measurement of Potential Soil Extracellular Enzyme Activities.” Journal of Visualized Experiments 81: 1–16. 10.3791/50961. PMC3991303 24299913

[emi470102-bib-0014] Bleuze, L. , G. Lashermes , G. Alavoine , S. Recous , and B. Chabbert . 2018. “Tracking the Dynamics of Hemp Dew Retting Under Controlled Environmental Conditions.” Industrial Crops and Products 123: 55–63. 10.1016/j.indcrop.2018.06.054.

[emi470102-bib-0016] Bou Orm, E. , A. Bergeret , and L. Malhautier . 2024a. “Microbial Communities and Their Role in Enhancing Hemp Fiber Quality Through Field Retting.” Applied Microbiology and Biotechnology 108: 501. 10.1007/s00253-024-13323-y. 39500773 PMC11538233

[emi470102-bib-0018] Bou Orm, E. , N. Sutton‐Charani , S. Bayle , J. C. Benezet , A. Bergeret , and L. Malhautier . 2024b. “Influence of Field Retting on Physicochemical and Biological Properties of “Futura 75” Hemp Stems.” Industrial Crops and Products 214: 118487. 10.1016/j.indcrop.2024.118487.

[emi470102-bib-0017] Bou Orm, E. , S. Sauvagère , J. Rocher , et al. 2023. “Estimating the Bias Related to DNA Recovery From Hemp Stems For Retting Microbial Community Investigation.” Applied Microbiology and Biotechnology 107: 4665–4681. 10.1007/s00253-023-12582-5. 37227475

[emi470102-bib-0019] Bourmaud, A. , D. U. Shah , J. Beaugrand , and H. N. Dhakal . 2020. “Property Changes in Plant Fibres During the Processing of Bio‐Based Composites.” Industrial Crops and Products 154: 112705. 10.1016/j.indcrop.2020.112705.

[emi470102-bib-0020] Brauman, A. , S. Kéléké , M. Malonga , E. Miambi , and F. Ampe . 1996. “Microbiological and Biochemical Characterization of Cassava Retting, a Traditional Lactic Acid Fermentation for Foo‐Foo (Cassava Flour) Production.” Applied and Environmental Microbiology 62: 2854–2858. 10.1128/aem.62.8.2854-2858.1996. 16535378 PMC1388916

[emi470102-bib-0021] Bredon, M. , J. Dittmer , C. Noël , B. Moumen , and D. Bouchon . 2018. “Lignocellulose Degradation at the Holobiont Level: Teamwork in a Keystone Soil Invertebrate.” Microbiome 6: 1–19. 10.1186/s40168-018-0536-y. 30223906 PMC6142342

[emi470102-bib-0022] Callahan, B. J. , P. J. McMurdie , M. J. Rosen , A. W. Han , A. J. A. Johnson , and S. P. Holmes . 2016. “DADA2: High‐Resolution Sample Inference From Illumina Amplicon Data.” Nature Methods 13, no. 7: 581–583. 10.1038/nmeth.3869. 27214047 PMC4927377

[emi470102-bib-0023] Chabbert, B. , J. Padovani , C. Djemiel , et al. 2020. “Multimodal Assessment of Flax Dew Retting and Its Functional Impact on Fibers and Natural Fiber Composites.” Industrial Crops and Products 148: 148. 10.1016/j.indcrop.2020.112255.

[emi470102-bib-0024] Challacombe, J. F. , C. N. Hesse , L. M. Bramer , et al. 2019. “Genomes and Secretomes of Ascomycota Fungi Reveal Diverse Functions in Plant Biomass Decomposition and Pathogenesis.” BMC Genomics 20: 1–27. 10.1186/s12864-019-6358-x. 31830917 PMC6909477

[emi470102-bib-0027] D'haeseleer, P. , J. M. Gladden , M. Allgaier , et al. 2013. “Proteogenomic Analysis of a Thermophilic Bacterial Consortium Adapted to Deconstruct Switchgrass.” PLoS One 8, no. 7: e68465. 10.1371/journal.pone.0068465. 23894306 PMC3716776

[emi470102-bib-0025] Datta, S. , D. Saha , L. Chattopadhyay , and B. Majumdar . 2020. “Genome Comparison Identifies Different Bacillus Species in a Bast Fibre‐Retting Bacterial Consortium and Provides Insights Into Pectin Degrading Genes.” Scientific Reports 10: 1–15. 10.1038/s41598-020-65228-1. 32424209 PMC7235092

[emi470102-bib-0026] DeAngelis, K. M. , M. Allgaier , Y. Chavarria , et al. 2011. “Characterization of Trapped Lignin‐Degrading Microbes in Tropical Forest Soil.” PLoS One 6, no. 4: e19306. 10.1371/journal.pone.0019306. 21559391 PMC3084812

[emi470102-bib-0028] Djemiel, C. , S. Dequiedt , B. Karimi , et al. 2022. “Potential of Meta‐Omics to Provide Modern Microbial Indicators for Monitoring Soil Quality and Securing Food Production.” Frontiers in Microbiology 13: 889788. 10.3389/fmicb.2022.889788. 35847063 PMC9280627

[emi470102-bib-0029] Djemiel, C. , S. Grec , and S. Hawkins . 2017. “Characterization of Bacterial and Fungal Community Dynamics by High‐Throughput Sequencing (HTS) Metabarcoding During Flax Dew‐Retting.” Frontiers in Microbiology 8: 2052. 10.3389/fmicb.2017.02052. 29104570 PMC5655573

[emi470102-bib-0030] Dong, X. , P. Gao , R. Zhou , C. Li , X. Dun , and X. Niu . 2021. “Changing Characteristics and Influencing Factors of the Soil Microbial Community During Litter Decomposition in a Mixed *Quercus Acutissima* Carruth. And *Robinia Pseudoacacia* L. Forest in Northern China.” Catena 196: 104811. 10.1016/j.catena.2020.104811.

[emi470102-bib-0032] Douglas, G. M. , V. J. Maffei , J. R. Zaneveld , et al. 2020. “PICRUSt2 for Prediction of Metagenome Functions.” Nature Biotechnology 38: 685–688. 10.1038/s41587-020-0548-6. PMC7365738 32483366

[emi470102-bib-0031] Douglas, G. M. , V. J. Maffei , J. Zaneveld , et al. 2019. “PICRUSt2: An Improved and Customizable Approach for Metagenome Inference.” 10.1101/672295.

[emi470102-bib-0033] Dumigan, C. R. , and M. K. Deyholos . 2024. “Soil and Seed Both Influence Bacterial Diversity in the Microbiome of the *Cannabis Sativa* Seedling Endosphere.” Frontiers in Plant Science 15: 1–13. 10.3389/fpls.2024.1326294. PMC10914941 38450399

[emi470102-bib-0034] Dzurendova, S. , C. B. Losada , B. X. Dupuy‐Galet , K. Fjær , and V. Shapaval . 2022. “ *Mucoromycota* Fungi as Powerful Cell Factories for Modern Biorefinery.” Applied Microbiology and Biotechnology 106: 101–115. 10.1007/s00253-021-11720-1. 34889982

[emi470102-bib-0035] Eichlerová, I. , L. Homolka , L. Žifčáková , L. Lisá , P. Dobiášová , and P. Baldrian . 2015. “Enzymatic Systems Involved in Decomposition Reflects the Ecology and Taxonomy of Saprotrophic Fungi.” Fungal Ecology 13: 10–22. 10.1016/j.funeco.2014.08.002.

[emi470102-bib-0036] Enarevba, D. R. , and K. R. Haapala . 2024. “The Emerging Hemp Industry: A Review of Industrial Hemp Materials and Product Manufacturing.” AgriEngineering 6: 2891–2925. 10.3390/agriengineering6030167.

[emi470102-bib-0037] Eren, M. I. , A. Chao , W. H. Hwang , and R. K. Colwell . 2012. “Estimating the Richness of a Population When the Maximum Number of Classes Is Fixed: A Nonparametric Solution to an Archaeological Problem.” PLoS One 7, no. 5: e34179. 10.1371/journal.pone.0034179. 22666316 PMC3362599

[emi470102-bib-0038] Feng, X. , H. Dong , P. Yang , et al. 2016. “Culture‐Dependent and Independent Methods to Investigate the Predominant Microorganisms Associated With Wet Processed Coffee.” Current Microbiology 73: 190–195. 10.1007/s00284-016-1047-3. 27113591

[emi470102-bib-0039] Foyer, C. H. , and G. Noctor . 2005. “Redox Homeostasis and Antioxidant Signaling: A Metabolic Interface Between Stress Perception and Physiological Responses.” Plant Cell 17: 1866–1875. 10.1105/tpc.105.033589. 15987996 PMC1167537

[emi470102-bib-0040] Fu, J. , H. Mueller , J. V. de Castro , et al. 2011. “Changes in the Bacterial Community Structure and Diversity During Bamboo Retting.” Biotechnology Journal 6: 1262–1271. 10.1002/biot.201100105. 21695788

[emi470102-bib-0041] Gacura, M. D. , D. D. Sprockett , B. Heidenreich , and C. B. Blackwood . 2016. “Comparison of Pectin‐Degrading Fungal Communities in Temperate Forests Using Glycosyl Hydrolase Family 28 Pectinase Primers Targeting Ascomycete Fungi.” Journal of Microbiological Methods 123: 108–113. 10.1016/j.mimet.2016.02.013. 26899925

[emi470102-bib-0042] Garbeva, P. , J. Postma , J. A. Van Veen , and J. D. Van Elsas . 2006. “Effect of Above‐Ground Plant Species on Soil Microbial Community Structure and Its Impact on Suppression of *Rhizoctonia Solani* AG3.” Environmental Microbiology 8: 233–246. 10.1111/j.1462-2920.2005.00888.x. 16423012

[emi470102-bib-0043] Gavande, P. V. , A. Basak , S. Sen , et al. 2021. “Functional Characterization of Thermotolerant Microbial Consortium for Lignocellulolytic Enzymes With Central Role of *Firmicutes* in Rice Straw Depolymerization.” Scientific Reports 11: 1–13. 10.1038/s41598-021-82163-x. 33542396 PMC7862241

[emi470102-bib-0045] Gilbert, H. J. 2010. “The Biochemistry and Structural Biology of Plant Cell Wall Deconstruction.” Plant Physiology 153: 444–455. 10.1104/pp.110.156646. 20406913 PMC2879781

[emi470102-bib-0046] Giraldo, A. , and P. W. Crous . 2019. “Inside Plectosphaerellaceae.” Studies in Mycology 92: 227–286. 10.1016/j.simyco.2018.10.005. 30518989 PMC6276054

[emi470102-bib-0047] Gladkov, G. V. , A. K. Kimeklis , A. M. Afonin , et al. 2022. “The Structure of Stable Cellulolytic Consortia Isolated From Natural Lignocellulosic Substrates.” International Journal of Molecular Sciences 23, no. 18: 10779. 10.3390/ijms231810779. 36142684 PMC9501375

[emi470102-bib-0050] Hasan, R. , N. Aktar , S. M. T. Kabir , et al. 2020. “Pectinolytic Bacterial Consortia Reduce Jute Retting Period and Improve Fibre Quality.” Scientific Reports 10: 1–9. 10.1038/s41598-020-61898-z. 32198430 PMC7083874

[emi470102-bib-0052] Hernández, R. , M. De Chaib Mares , H. Jimenez , A. Reyes , and A. Caro‐Quintero . 2022. “Functional and Phylogenetic Characterization of Bacteria in Bovine Rumen Using Fractionation of Ruminal Fluid.” Frontiers in Microbiology 13: 1–17. 10.3389/fmicb.2022.813002. PMC8992543 35401437

[emi470102-bib-0053] Hu, X. , J. Yu , C. Wang , and H. Chen . 2014. “Cellulolytic Bacteria Associated With the Gut of *Dendroctonus Armandi* Larvae (*Coleoptera: Curculionidae: Scolytinae*).” Forests 5: 455–465. 10.3390/f5030455.

[emi470102-bib-0054] Ivanova, A. A. , C. E. Wegner , Y. Kim , W. Liesack , and S. N. Dedysh . 2016. “Identification of Microbial Populations Driving Biopolymer Degradation in Acidic Peatlands by Metatranscriptomic Analysis.” Molecular Ecology 25: 4818–4835. 10.1111/mec.13806. 27545292

[emi470102-bib-0055] Ji, L. , J. Yang , H. Fan , et al. 2014. “Synergy of Crude Enzyme Cocktail From Cold‐Adapted *Cladosporium Cladosporioides* Ch2‐2 With Commercial Xylanase Achieving High Sugars Yield at Low Cost.” Biotechnology for Biofuels 7: 1–12. 10.1186/s13068-014-0130-x. 25254072 PMC4172917

[emi470102-bib-0056] Ke, P. J. , T. Miki , and T. S. Ding . 2015. “The Soil Microbial Community Predicts the Importance of Plant Traits in Plant‐Soil Feedback.” New Phytologist 206: 329–341. 10.1111/nph.13215. 25521190

[emi470102-bib-0057] Khalaf, E. M. , and M. N. Raizada . 2020. “Draft Genome Sequences of *Pantoea agglomerans* , *Paenibacillus Polymyxa*, and *Pseudomonas sp*. Strains, Seed Biogel‐Associated Endophytes of *Cucumis sativus* L. (Cucumber) and *Cucumis melo* L. (Cantaloupe).” Microbiology Resource Announcements 32: e00667. 10.1128/MRA.00667-20. PMC7409851 32763934

[emi470102-bib-0058] Koivula, M. J. , H. R. Kymäläinen , L. Vanne , S. Levo , A. Pehkonen , and A. M. Sjöberg . 2004. “Microbial Quality of Linseed and Fibre Hemp Plants During Growing and Harvest Seasons.” Agriculture and Food Science 13: 327–337. 10.2137/1239099043633369.

[emi470102-bib-0059] Kumah, E. A. , R. D. Fopa , S. Harati , P. Boadu , F. V. Zohoori , and T. Pak . 2023. “Human and Environmental Impacts of Nanoparticles: A Scoping Review of the Current Literature.” BMC Public Health 23, no. 1: 1059. 10.1186/s12889-023-15958-4. 37268899 PMC10239112

[emi470102-bib-0060] Kurm, V. , S. Geisen , and W. H. Gera Hol . 2019. “A Low Proportion of Rare Bacterial Taxa Responds to Abiotic Changes Compared With Dominant Taxa.” Environmental Microbiology 21: 750–758. 10.1111/1462-2920.14492. 30507058 PMC7379498

[emi470102-bib-0061] Lacerda, G. V. , M. F. Noronha , S. T. P. de Sousa , et al. 2017. “Potential of Semiarid Soil From Caatinga Biome as a Novel Source for Mining Lignocellulose‐Degrading Enzymes.” FEMS Microbiology Ecology 93, no. 2: 1–15. 10.1093/femsec/fiw248. 27986827

[emi470102-bib-0062] Langille, M. G. I. , J. Zaneveld , J. G. Caporaso , et al. 2013. “Predictive Functional Profiling of Microbial Communities Using 16S rRNA Marker Gene Sequences.” Nature Biotechnology 31: 814–821. 10.1038/nbt.2676. PMC3819121 23975157

[emi470102-bib-0064] Law, A. D. , C. R. McNees , and L. A. Moe . 2020. “The Microbiology of Hemp Retting in a Controlled Environment: Steering the Hemp Microbiome Towards More Consistent Fiber Production.” Agronomy 10: 1–10. 10.3390/agronomy10040492.

[emi470102-bib-0065] Le Duigou, A. , J. M. Deux , P. Davies , and C. Baley . 2011. “Protection of Flax/PLLA Biocomposites From Seawater Ageing by External Layers of PLLA.” International Journal of Polymeric Science 2011: 1–8. 10.1155/2011/235805.

[emi470102-bib-0066] Lee, C. H. , A. Khalina , S. H. Lee , and M. Liu . 2020. “A Comprehensive Review on Bast Fibre Retting Process for Optimal Performance in Fibre‐Reinforced Polymer Composites.” Advances in Materials Science and Engineering 2020, no. 1: 6074063. 10.1155/2020/6074063.

[emi470102-bib-0067] Lelievre, M. 2020. “Mesure de la Biomasse Moléculaire Microbienne du Sol.” PARTIE 1: Extraction d'ADN Brut du Sol. https://www2.dijon.inrae.fr/plateforme_genosol/.

[emi470102-bib-0068] Li, S. H. , S. L. Feng , Z. T. Li , et al. 2011. “Isolation, Identification and Characterization of Oleaginous Fungi From the Soil of Qinghai Plateau That Utilize D‐Xylose.” African Journal of Microbiology Research 5: 2075–2081. 10.5897/ajmr11.171.

[emi470102-bib-0069] Li, T. , D. Shi , Q. Wu , et al. 2019. “Mechanism of Cell Wall Polysaccharides Modification in Harvested ‘Shatangju’ Mandarin (Citrus Reticulate Blanco) Fruit Caused by *Penicillium Italicum* .” Biomolecules 9: 9. 10.3390/biom9040160. PMC6523094 31022937

[emi470102-bib-0070] Li, Y. J. , C. H. Chuang , W. C. Cheng , et al. 2022. “A Metagenomics Study of Hexabromocyclododecane Degradation With a Soil Microbial Community.” Journal of Hazardous Materials 430: 128465. 10.1016/j.jhazmat.2022.128465. 35739659

[emi470102-bib-0071] Lindahl, B. , and J. Boberg . 2008. “Chapter 10 Distribution and Function of Litter *Basidiomycetes* in Coniferous Forests.” In Br Mycol Soc Symp Series, vol. 28, 183–196. Elsevier.

[emi470102-bib-0072] Liu, H. , F. Pan , X. Han , et al. 2019. “Response of Soil Fungal Community Structure to Long‐Term Continuous Soybean Cropping.” Frontiers in Microbiology 9: 1–13. 10.3389/fmicb.2018.03316. PMC6333693 30687292

[emi470102-bib-0073] Liu, M. , M. T. Ale , B. Kołaczkowski , et al. 2017. “Comparison of Traditional Field Retting and *Phlebia Radiata Cel 26* Retting of Hemp Fibres for Fibre‐Reinforced Composites.” AMB Express 7, no. 1: 58. 10.1186/s13568-017-0355-8. 28275995 PMC5342995

[emi470102-bib-0075] Logares, R. , E. S. Lindström , S. Langenheder , et al. 2013. “Biogeography of Bacterial Communities Exposed to Progressive Long‐Term Environmental Change.” ISME Journal 7: 937–948. 10.1038/ismej.2012.168. 23254515 PMC3635229

[emi470102-bib-0076] López‐Mondéjar, R. , V. Tláskal , U. N. da Rocha , and P. Baldrian . 2022. “Global Distribution of Carbohydrate Utilization Potential in the Prokaryotic Tree of Life.” MSystems 7, no. 6: e0082922. 10.1128/msystems.00829-22. 36413015 PMC9765126

[emi470102-bib-0077] Louca, S. , M. Doebeli , and L. W. Parfrey . 2018. “Correcting for 16S rRNA Gene Copy Numbers in Microbiome Surveys Remains an Unsolved Problem.” Microbiome 6: 1–12. 10.1186/s40168-018-0420-9. 29482646 PMC5828423

[emi470102-bib-0078] Lynd, L. R. , P. J. Weimer , W. H. van Zyl , and S. Isak . 2002. “Microbial Cellulose Utilization: Fundamentals and Biotechnology.” Microbiology and Molecular Biology Reviews 66: 506–577. 10.1128/MMBR.66.3.506. 12209002 PMC120791

[emi470102-bib-0079] Ma, C. , X. Chen , G. Zheng , N. Liu , J. Zhao , and H. Zhang . 2022. “Exploring the Influence Mechanisms of Polystyrene‐Microplastics on Sewage Sludge Composting.” Bioresource Technology 362: 127798. 10.1016/j.biortech.2022.127798. 35995344

[emi470102-bib-0080] Ma, R. , H. Huang , Y. Bai , H. Luo , Y. Fan , and B. Yao . 2018. “Insight Into the Cold Adaptation and Hemicellulose Utilization of *Cladosporium Neopsychrotolerans* From Genome Analysis and Biochemical Characterization.” Scientific Reports 8: 1–14. 10.1038/s41598-018-24443-7. 29666397 PMC5904165

[emi470102-bib-0082] Marcon, E. 2010. Mesures de la Biodiversité, 58.

[emi470102-bib-0083] Mazian, B. , A. Bergeret , J. C. Benezet , and L. Malhautier . 2018. “Influence of Field Retting Duration on the Biochemical, Microstructural, Thermal and Mechanical Properties of Hemp Fibres Harvested at the Beginning of Flowering.” Industrial Crops and Products 116: 170–181. 10.1016/j.indcrop.2018.02.062.

[emi470102-bib-0084] Mazian, B. , A. Bergeret , J. C. Benezet , and L. Malhautier . 2020. “Impact of Field Retting and Accelerated Retting Performed in a Lab‐Scale Pilot Unit on the Properties of Hemp Fibres/Polypropylene Biocomposites.” Industrial Crops and Products 143: 111912. 10.1016/j.indcrop.2019.111912.

[emi470102-bib-0085] Mazian, B. , S. Cariou , M. Chaignaud , et al. 2019. “Evolution of Temporal Dynamic of Volatile Organic Compounds (VOCs) and Odors of Hemp Stem During Field Retting.” Planta 250: 1983–1996. 10.1007/s00425-019-03280-6. 31529396

[emi470102-bib-0086] McMurdie, P. J. , and S. Holmes . 2013. “Phyloseq: An R Package for Reproducible Interactive Analysis and Graphics of Microbiome Census Data.” PLoS One 8, no. 4: e61217. 10.1371/journal.pone.0061217. 23630581 PMC3632530

[emi470102-bib-0087] Merín, M. G. , L. M. Mendoza , V. Inés , and V. I. M. de Ambrosini . 2014. “Pectinolytic Yeasts From Viticultural and Enological Environments: Novel Finding of *Filobasidium Capsuligenum* Producing Pectinases.” Journal of Basic Microbiology 54, no. 8: 835–842. 10.1002/jobm.201200534. 23686851

[emi470102-bib-0088] Moawad, H. , W. M. Abd El‐Rahim , M. M. Hashem , et al. 2019. “Retting and Degumming of Flax Using Biotechnology Eco‐Friendly Approach.” Egyptian Journal of Chemistry 62: 2033–2045. 10.21608/EJCHEM.2019.9571.1641.

[emi470102-bib-0089] Moser, L. E. 1995. “Post‐Harvest Physiological Changes in Forage Plants.” In Post‐Harvest Physiol Preserv Forages, 1–19. American Society of Agronomy, Crop Science Society of America, and Soil Science Society of America. 10.2135/cssaspecpub22.c1.

[emi470102-bib-0090] Mukherjee, S. , E. Goulas , A. Creach , et al. 2024. “Metaproteomics Identifies Key Cell Wall Degrading Enzymes and Proteins Potentially Related to Inter‐Field Variability in Fiber Quality During Flax Dew Retting.” Industrial Crops and Products 222: 222. 10.1016/j.indcrop.2024.119907.

[emi470102-bib-0091] Munshi, T. K. , and B. B. Chattoo . 2008. “Bacterial Population Structure of the Jute‐Retting Environment.” Microbial Ecology 56: 270–282. 10.1007/s00248-007-9345-8. 18097714

[emi470102-bib-0092] Murali, A. , B. Aniruddha , and S. W. Erik . 2018. “IDTAXA: A Novel Approach for Accurate Taxonomic Classification of Microbiome Sequences.” Microbiome 6, no. 1: 140. 10.1186/s40168-018-0521-5. 30092815 PMC6085705

[emi470102-bib-0151] Murphy, C. L. , R. Yang , T. Decker , et al. 2021. “Genomes of Novel Myxococcota Reveal Severely Curtailed Machineries for Predation and C0ellular Differentiation.” Applied and Environmental Microbiology 87, no. 23: e01706‐21. 10.1128/AEM.01706-21. 34524899 PMC8580003

[emi470102-bib-0093] Mushimiyimana, I. , and P. Tallapragada . 2013. “Optimization of Process Parameters for Biosynthesis of Cellulase by *Cladosporium Cladosporioides* Using Agro Wastes.” International Journal of Pharma and Bio Sciences 4: 1129–1138.

[emi470102-bib-0094] Müssig, J. 2010. Industrial Applications of Natural Fibres: Structure, Properties and Technical Applications, 536. Wiley.

[emi470102-bib-0096] O'Hara, R. B. 2005. “Species Richness Estimators: How Many Species Can Dance on the Head of a Pin?” Journal of Animal Ecology 74: 375–386. 10.1111/j.1365-2656.2005.00940.x.

[emi470102-bib-0097] Okolie, J. A. , S. Nanda , A. K. Dalai , and J. A. Kozinski . 2021. “Chemistry and Specialty Industrial Applications of Lignocellulosic Biomass.” Waste and Biomass Valorization 12: 2145–2169. 10.1007/s12649-020-01123-0.

[emi470102-bib-0098] Pandit, P. D. , M. K. Gulhane , A. A. Khardenavis , and H. J. Purohit . 2016. “Mining of Hemicellulose and Lignin Degrading Genes From Differentially Enriched Methane Producing Microbial Community.” Bioresource Technology 216: 923–930. 10.1016/j.biortech.2016.06.021. 27323244

[emi470102-bib-0099] Parks, D. H. , M. Chuvochina , C. Rinke , A. J. Mussig , P. A. Chaumeil , and P. Hugenholtz . 2022. “GTDB: An Ongoing Census of Bacterial and Archaeal Diversity Through a Phylogenetically Consistent, Rank Normalized and Complete Genome‐Based Taxonomy.” Nucleic Acids Research 50: D785–D794. 10.1093/nar/gkab776. 34520557 PMC8728215

[emi470102-bib-0100] Perkins, A. K. , A. L. Rose , H. P. Grossart , K. Rojas‐Jimenez , S. K. Barroso Prescott , and J. M. Oakes . 2021. “Oxic and Anoxic Organic Polymer Degradation Potential of Endophytic Fungi From the Marine Macroalga, Ecklonia Radiata.” Frontiers in Microbiology 12: 1–13. 10.3389/fmicb.2021.726138. PMC8558676 34733248

[emi470102-bib-0101] Placet, V. , A. Day , and J. Beaugrand . 2017. “The Influence of Unintended Field Retting on the Physicochemical and Mechanical Properties of Industrial Hemp Bast Fibres.” Journal of Materials Science 52: 5759–5777. 10.1007/s10853-017-0811-5.

[emi470102-bib-0102] Purahong, W. , T. Wubet , G. Lentendu , et al. 2016. “Life in Leaf Litter: Novel Insights Into Community Dynamics of Bacteria and Fungi During Litter Decomposition.” Molecular Ecology 25: 4059–4074. 10.1111/mec.13739. 27357176

[emi470102-bib-0103] Quast, C. , P. Elmar , Y. Pelin , et al. 2013. “The SILVA Ribosomal RNA Gene Database Project: Improved Data Processing and Web‐Based Tools.” Nucleic Acids Research 41, no. Database issue: 590–596. 10.1093/nar/gks1219. PMC3531112 23193283

[emi470102-bib-0104] Rafique, N. , R. Tabassum , M. S. Awan , W. Orts , and D. W. S. Wong . 2016. “Cloning and Expression of *Pectobacterium Carotovorum* Endo‐Polygalacturonase Gene in *Pichia Pastoris* for Production of Oligogalacturonates.” BioResources 11: 5204–5214. 10.15376/biores.11.2.5204-5214.

[emi470102-bib-0105] Ralph, J. , K. Lundquist , G. Brunow , et al. 2004. “Lignins: Natural Polymers From Oxidative Coupling of 4‐Hydroxyphenyl‐ Propanoids.” Phytochemistry Reviews 3: 29–60. 10.1023/B:PHYT.0000047809.65444.a4.

[emi470102-bib-0106] Rehman, M. , S. Fahad , G. Du , et al. 2021. “Evaluation of Hemp ( *Cannabis sativa* L.) as an Industrial Crop: A Review.” Environmental Science and Pollution Research 28: 52832–52843. 10.1007/s11356-021-16264-5. 34476693

[emi470102-bib-0107] Renier, A. , S. M. De Faria , P. Jourand , et al. 2011. “Nodulation of *Crotalaria Podocarpa* DC. By *Methylobacterium nodulans* Displays Very Unusual Features.” Journal of Experimental Botany 62: 3693–3697. 10.1093/jxb/err083. 21422120

[emi470102-bib-0108] Réquilé, S. , B. Mazian , M. Grégoire , et al. 2021. “Exploring the Dew Retting Feasibility of Hemp in Very Contrasting European Environments: Influence on the Tensile Mechanical Properties of Fibres and Composites.” Industrial Crops and Products 164: 113337. 10.1016/j.indcrop.2021.113337.

[emi470102-bib-0109] Reynolds, N. K. , J. E. Stajich , G. L. Benny , et al. 2023. “Mycoparasites, Gut Dwellers, and Saprotrophs: Phylogenomic Reconstructions and Comparative Analyses of Kickxellomycotina Fungi.” Genome Biology and Evolution 15: 1–22. 10.1093/gbe/evac185. PMC9866270 36617272

[emi470102-bib-0110] Ribeiro, A. , P. Pochart , A. Day , et al. 2015. “Microbial Diversity Observed During Hemp Retting.” Applied Microbiology and Biotechnology 99: 4471–4484. 10.1007/s00253-014-6356-5. 25575888

[emi470102-bib-0111] Richely, E. , A. Bourmaud , V. Placet , S. Guessasma , and J. Beaugrand . 2022. “A Critical Review of the Ultrastructure, Mechanics and Modelling of Flax Fibres and Their Defects.” Progress in Materials Science 124: 100851. 10.1016/j.pmatsci.2021.100851.

[emi470102-bib-0112] Rifa, E. , and S. Theil . 2010. “ExploreMetabar: v2.0.1.” Zenodo. https://forgemia.inra.fr/umrf/exploremetabar.

[emi470102-bib-0113] Rytioja, J. , K. Hildén , J. Yuzon , A. Hatakka , R. P. de Vries , and M. R. Mäkelä . 2014. “Plant‐Polysaccharide‐Degrading Enzymes From *Basidiomycetes* .” Microbiology and Molecular Biology Reviews 78: 614–649. 10.1128/mmbr.00035-14. 25428937 PMC4248655

[emi470102-bib-0114] Sahoo, R. K. , M. Gaur , A. Das , A. Singh , M. Kumar , and E. Subudhi . 2017. “Comparative Analysis of 16S rRNA Gene Illumina Sequence for Microbial Community Structure in Diverse Unexplored Hot Springs of Odisha, India.” Geomicrobiology Journal 34: 567–576. 10.1080/01490451.2016.1238980.

[emi470102-bib-0115] Sauvadet, M. , M. Chauvat , D. Cluzeau , P. A. Maron , C. Villenave , and I. Bertrand . 2016. “The Dynamics of Soil Micro‐Food Web Structure and Functions Vary According to Litter Quality.” Soil Biology and Biochemistry 95: 262–274. 10.1016/j.soilbio.2016.01.003.

[emi470102-bib-0116] Schellenberger, S. , S. Kolb , and H. L. Drake . 2010. “Metabolic Responses of Novel Cellulolytic and Saccharolytic Agricultural Soil Bacteria to Oxygen.” Environmental Microbiology 12: 845–861. 10.1111/j.1462-2920.2009.02128.x. 20050868

[emi470102-bib-0117] Schlaepfer, R. , and R. Bütler . 2002. “Analyse de la Dynamique du Paysage, Fiche D'enseignement 4.2.” École Polytechnique de Lausanne.

[emi470102-bib-0118] Senthilguru, K. , T. S. George , N. S. Vasanthi , and K. P. Kannan . 2011. “Ethanol Production From Lignocellulosic Waste.” World Journal of Science and Technology 1: 12–16.

[emi470102-bib-0119] Shade, A. , S. E. Jones , J. Gregory Caporaso , et al. 2014. “Conditionally Rare Taxa Disproportionately Contribute to Temporal Changes in Microbial Diversity.” MBio 5, no. 4: e01371. 10.1128/mBio.01371-14. 25028427 PMC4161262

[emi470102-bib-0120] Sharma, H. S. S. 1986. “An Alternative Method of Flax Retting During Dry Weather.” Annals of Applied Biology 109: 605–611. 10.1111/j.1744-7348.1986.tb03217.x.

[emi470102-bib-0121] Simpson, E. 1949. “Measurement of Diversity.” Nature 163: 688. 10.1038/163688a0.

[emi470102-bib-0122] Sista Kameshwar, A. K. , and W. Qin . 2019. “Systematic Review of Publicly Available Non‐Dikarya Fungal Proteomes for Understanding Their Plant Biomass‐Degrading and Bioremediation Potentials.” Bioresource Technology 6, no. 1: 6. 10.1186/s40643-019-0264-6.

[emi470102-bib-0123] Sisti, L. , G. Totaro , M. Vannini , and A. Celli . 2018. “Retting Process as a Pretreatment of Natural Fibers for the Development of Polymer Composites.” In Lignocellulosic Composite Materials, 97–135. Springer International Publishing AG. 10.1007/978-3-319-68696-7_2.

[emi470102-bib-0124] Song, N. , H. Xu , Z. Yan , T. Yang , C. Wang , and H. L. Jiang . 2019. “Improved Lignin Degradation Through Distinct Microbial Community in Subsurface Sediments of One Eutrophic Lake.” Renewable Energy 138: 861–869. 10.1016/j.renene.2019.01.121.

[emi470102-bib-0125] Speight, J. G. 2020. “Non–Fossil Fuel Feedstocks.” In The Refnery of the Future, 2nd ed., 343–389. Elsevier (Gulf Professional Publishing).

[emi470102-bib-0126] Štursová, M. , L. Žifčáková , M. B. Leigh , R. Burgess , and P. Baldrian . 2012. “Cellulose Utilization in Forest Litter and Soil: Identification of Bacterial and Fungal Decomposers.” FEMS Microbiology Ecology 80: 735–746. 10.1111/j.1574-6941.2012.01343.x. 22379979

[emi470102-bib-0127] Tao, J. , Q. Chen , S. Chen , et al. 2022. “Metagenomic Insight Into the Microbial Degradation of Organic Compounds in Fermented Plant Leaves.” Environmental Research 214: 113902. 10.1016/j.envres.2022.113902. 35839908

[emi470102-bib-0128] Ten, E. , and W. Vermerris . 2013. “Functionalized Polymers From Lignocellulosic Biomass: State of the Art.” Polymers (Basel) 5: 600–642.

[emi470102-bib-0129] Theil, S. , and E. Rifa . 2021. “RANOMALY: AmplicoN WOrkflow for Microbial Community AnaLYsis.” F1000Research 10, no. 7: 7. 10.12688/f1000research.27268.1. 33537122 PMC7836088

[emi470102-bib-0130] Tian, B. Y. , Q. G. Huang , Y. Xu , C. X. Wang , R. R. Lv , and J. Z. Huang . 2010. “Microbial Community Structure and Diversity in a Native Forest Wood‐Decomposed Hollow‐Stump Ecosystem.” World Journal of Microbiology and Biotechnology 26: 233–240. 10.1007/s11274-009-0165-5.

[emi470102-bib-0131] Tom, L. M. , M. Aulitto , Y. W. Wu , et al. 2022. “Low‐Abundance Populations Distinguish Microbiome Performance in Plant Cell Wall Deconstruction.” Microbiome 10: 1–16. 10.1186/s40168-022-01377-x. 36280858 PMC9594917

[emi470102-bib-0132] Urquhart, E. J. , and Z. K. Punja . 2002. “Hydrolytic Enzymes and Antifungal Compounds Produced by Tilletiopsis Species, Phyllosphere Yeasts That Are Antagonists of Powdery Mildew Fungi.” Canadian Journal of Microbiology 48: 219–229. 10.1139/w02-008. 11989766

[emi470102-bib-0133] Ventorino, V. , A. Aliberti , V. Faraco , et al. 2015. “Exploring the Microbiota Dynamics Related to Vegetable Biomasses Degradation and Study of Lignocellulose‐Degrading Bacteria for Industrial Biotechnological Application.” Scientific Reports 5: 8161. 10.1038/srep08161. 25641069 PMC4648445

[emi470102-bib-0134] Visi, D. K. , N. D'Souza , B. G. Ayre , C. L. Webber , and M. S. Allen . 2013. “Investigation of the Bacterial Retting Community of Kenaf (*Hibiscus Cannabinus*) Under Different Conditions Using Next‐Generation Semiconductor Sequencing.” Journal of Industrial Microbiology & Biotechnology 40: 465–475. 10.1007/s10295-013-1242-1. 23475284

[emi470102-bib-0135] Voriskova, J. , and P. Baldrian . 2013. “Fungal Community on Decomposing Leaf Litter Undergoes Rapid Successional Changes.” ISME Journal 7: 477–486. 10.1038/ismej.2012.116. 23051693 PMC3578564

[emi470102-bib-0136] Wang, C. , D. Dong , H. Wang , et al. 2016. “Metagenomic Analysis of Microbial Consortia Enriched From Compost: New Insights Into the Role of *Actinobacteria* in Lignocellulose Decomposition.” Biotechnology for Biofuels 9: 1–17. 10.1186/s13068-016-0440-2. 26834834 PMC4731972

[emi470102-bib-0137] Wang, Y. , L. Ji , Q. Li , et al. 2021b. “Effects of Long‐Term Bare Fallow During the Winter‐Wheat Growth Season on the Soil Chemical Properties, Fungal Community Composition, and the Occurrence of Maize Fungal Diseases in North China.” Plant Disease 105: 2575–2584. 10.1094/PDIS-11-20-2492-RE. 33404273

[emi470102-bib-0138] Wang, Y. , T. Jin , N. Zhang , et al. 2021a. “Effect of Stocking Density and Age on Physiological Performance and Dynamic Gut Bacterial and Fungal Communities in Langya Hens.” Microbial Cell Factories 20: 1–15. 10.1186/s12934-021-01707-y. 34863176 PMC8642922

[emi470102-bib-0139] Ware, I. M. , M. E. Van Nuland , Z. K. Yang , C. W. Schadt , J. A. Schweitzer , and J. K. Bailey . 2021. “Climate‐Driven Divergence in Plant‐Microbiome Interactions Generates Range‐Wide Variation in Bud Break Phenology.” Communications Biology 4: 1–9. 10.1038/s42003-021-02244-5. 34135464 PMC8209103

[emi470102-bib-0140] Woo, H. L. , T. C. Hazen , B. A. Simmons , and K. M. DeAngelis . 2014. “Enzyme Activities of Aerobic Lignocellulolytic Bacteria Isolated From Wet Tropical Forest Soils.” Systematic and Applied Microbiology 37: 60–67. 10.1016/j.syapm.2013.10.001. 24238986

[emi470102-bib-0141] Xu, H. , L. Zhang , X. Feng , et al. 2022. “Metagenomic and Proteomic Analysis of Bacterial Retting Community and Proteome Profile in the Degumming Process of Kenaf Bast.” BMC Plant Biology 22: 1–11. 10.1186/s12870-022-03890-5. 36333799 PMC9636830

[emi470102-bib-0142] Yu, M. , J. Zhang , Y. Xu , et al. 2015. “Fungal Community Dynamics and Driving Factors During Agricultural Waste Composting.” Environmental Science and Pollution Research 22: 19879–19886. 10.1007/s11356-015-5172-5. 26289327

[emi470102-bib-0144] Zainudin, M. H. M. , M. A. Hassan , M. Tokura , and Y. Shirai . 2013. “Indigenous Cellulolytic and Hemicellulolytic Bacteria Enhanced Rapid Co‐Composting of Lignocellulose Oil Palm Empty Fruit Bunch With Palm Oil Mill Effluent Anaerobic Sludge.” Bioresource Technology 147: 632–635. 10.1016/j.biortech.2013.08.061. 24012093

[emi470102-bib-0145] Zhang, C. , B. Cai , Y. Sun , J. Kang , F. Pei , and J. Ge . 2023. “Microbial Communities That Drive the Degradation of Flax Pectin and Hemicellulose During Dew Retting With *Bacillus licheniformis* HDYM‐04 and *Bacillus subtilis* ZC‐01 Addition.” Bioresource Technology 371: 128516. 10.1016/j.biortech.2022.128516. 36563865

[emi470102-bib-0146] Zhang, T. , Z. Wang , X. Lv , Y. Li , and L. Zhuang . 2019. “High‐Throughput Sequencing Reveals the Diversity and Community Structure of Rhizosphere Fungi of *Ferula Sinkiangensis* at Different Soil Depths.” Scientific Reports 9: 1–10. 10.1038/s41598-019-43110-z. 31024051 PMC6484027

[emi470102-bib-0147] Zhao, D. , P. Liu , C. Pan , R. Du , W. Ping , and J. Ge . 2016. “Bacterial Succession and Metabolite Changes During Flax (*Linum Usitatissimum* L.) Retting With *Bacillus Cereus* HDYM‐02.” Scientific Reports 6: 1–9. 10.1038/srep31812. 27585559 PMC5009381

[emi470102-bib-0148] Zhou, L. , X. Yang , Y. Xie , et al. 2022. “The Effect of Bacteria Inoculation on the Lignocellulose Degradation and the Microbial Properties During Cow Dung Composting.” Research Square. 10.21203/rs.3.rs-1541181/v1. PMC10599258 37471462

[emi470102-bib-0149] Zhu, W. L. , J. Y. Cui , L. Y. Cui , et al. 2016. “Bioconversion of Methanol to Value‐Added Mevalonate by Engineered *Methylobacterium extorquens* AM1 Containing an Optimized Mevalonate Pathway.” Applied Microbiology and Biotechnology 100: 2171–2182. 10.1007/s00253-015-7078-z. 26521242

[emi470102-bib-0150] Zimniewska, M. 2022. “Hemp Fibre Properties and Processing Target Textile: A Review.” Materials (Basel) 15: 1901. 10.3390/ma15051901. 35269132 PMC8911747

