# Correction to ‘Enhancing Biodiversity‐Function Relationships in Field Retting: Towards Key Microbial Indicators for Retting Control’

**DOI:** 10.1111/1758-2229.70334

**Published:** 2026-04-05

Bou Orm, E., S. Mukherjee, E. Rifa, et al. 2025. “Enhancing Biodiversity‐Function Relationships in Field Retting: Towards Key Microbial Indicators for Retting Control.” *Environmental Microbiology Reports* 17, no. 3: e70102. https://doi.org/10.1111/1758‐2229.70102.

In the above article, we found an error in the citations of figure numbers. The correct information is shown below for reference:

- Page 5 of 24, §3.2. Microbial Community Diversity: **Figure S7** should be **Figure 1**.
- Page 6 of 24, §3.2. Microbial Community Diversity:
**Figure S9, S10** should only be **Figure S9**.
**Figure 1** should be **Figure 2**.
**Figure 1A** should be **Figure 2A** within the paragraph ‘The bacterial community analysis (Figure 1A) reveals…indicating a clustering at R4 and R6 (circle in Figure 1A)’.
**Figure 1B** should be **Figure 2B**.
**Figure 2** should be **Figure 3**.
- Page 9 of 24, §3.3. Community Structure:
**Figure 3** should be **Figure 4**.
**Figure 4** should be **Figure 5**.
- Page 10 of 24, §3.3. Community Structure: **Figure 5** should be **Figure 6**.
- Page 11 of 24, §3.4. Prediction of Potential Bacterial Enzymatic: **Figure 6, Figure 6D, Figure 6C, Figure 6B** should be **Figure 7, Figure 7D, Figure 7C, Figure 7B** respectively.
- Page 12 of 24, §3.5. Evolution of Enzyme Expression During Retting:
**Figure 7, Figure 7A, Figure 7B, Figure 7C** should be **Figure 8, Figure 8A, Figure 8B, Figure 8C** respectively.
**Figure 8** should be **Figure S7**.

We apologise for this error.

