# Supplementary material for: Enhancing Biodiversity‐Function Relationships in Field Retting: Towards Key Microbial Indicators for Retting Control
Source: Environ Microbiol Rep. 2025 Jun 18;17(3):e70102. doi: 10.1111/1758-2229.70102 (PMC12175878; doi:10.1111/1758-2229.70102)
Supplement: Supplementary file 1 — Figure S1. (A) Sampling sites: Drôme Chanvre, Mirabel et Blacons, France (green icon), laboratory site, Alès, France (grey icon), and Mas de la Valus, Bouquet, France (red icon). (B) The orange circle indicates the location of the retting site relative to the country map (France). The maps are generated on OpenStreetMap. Figure S2. Experimental site during the field retting campaign. The numbers correspond to the distance (in meters) between swaths. Figure S3. Photograph of the pilot unit illustrating the laboratory‐scale retting process. The pilot unit is filled with gravel/pozzolan (10 cm high) (1) covered by a layer of geotextile (2), 15 cm of soil (3), and the hemp stems (4). Figure S4. Sampling plan used during the retting campaign in the field and in the pilot unit. Figure S5. Rarefaction curves corresponding to bacterial (16S) and fungal (18S) domains for all samples. Figure S6. Venn Diagram illustrating the shared bacterial (A) and fungal (B) ASV among swaths 1, 3, and 4 retted in the field. Figure S7.Venn diagram of all common bacterial (A) and fungal (B) ASV between soil and stem samples (R0 and R4) belonging to the swath 4 retted in the field. Figure S8. Venn diagram of all common bacterial (A) and fungal (B) ASV between soil field and soil of the pilot unit samples (R0 and R2). Figure S9. Principal coordinates analysis (PCoA) of bacterial (16S) (A) and fungal (18S) (B) community structure for field and pilot unit stem and soil samples. Figure S10. Bacterial relative abundance at phylum (A) and class (B) levels in unretted and retted stem samples (swaths 1, 3, and 4) in the field over time. R0: unretted samples, R1, R2, R3, R4, and R6 correspond to retted samples after 1, 2, 3, 4, and 6 weeks of retting. Figure S11. Fungal relative abundance at phylum (A) and class (B) levels in unretted and retted stem samples (swaths 1, 3, and 4) in the field over time. R0: unretted samples, R1, R2, R3, R4, and R6 correspond to retted samples after 1, 2, 3, 4, and 6 wee [file EMI4-17-e70102-s002.docx]

Supplementary Material

**Sequencing reveals microbial dynamics of hemp field retting**

**Eliane Bou Orm*, Suvajit Mukherjee, Etienne Rifa, Anne Créach, Sébastien Grec, Sandrine Bayle, Jean-Charles Benezet, Anne Bergeret, Luc Malhautier**

*** Correspondence:** Corresponding Author: eliane.bou-orm@uha.fr

**
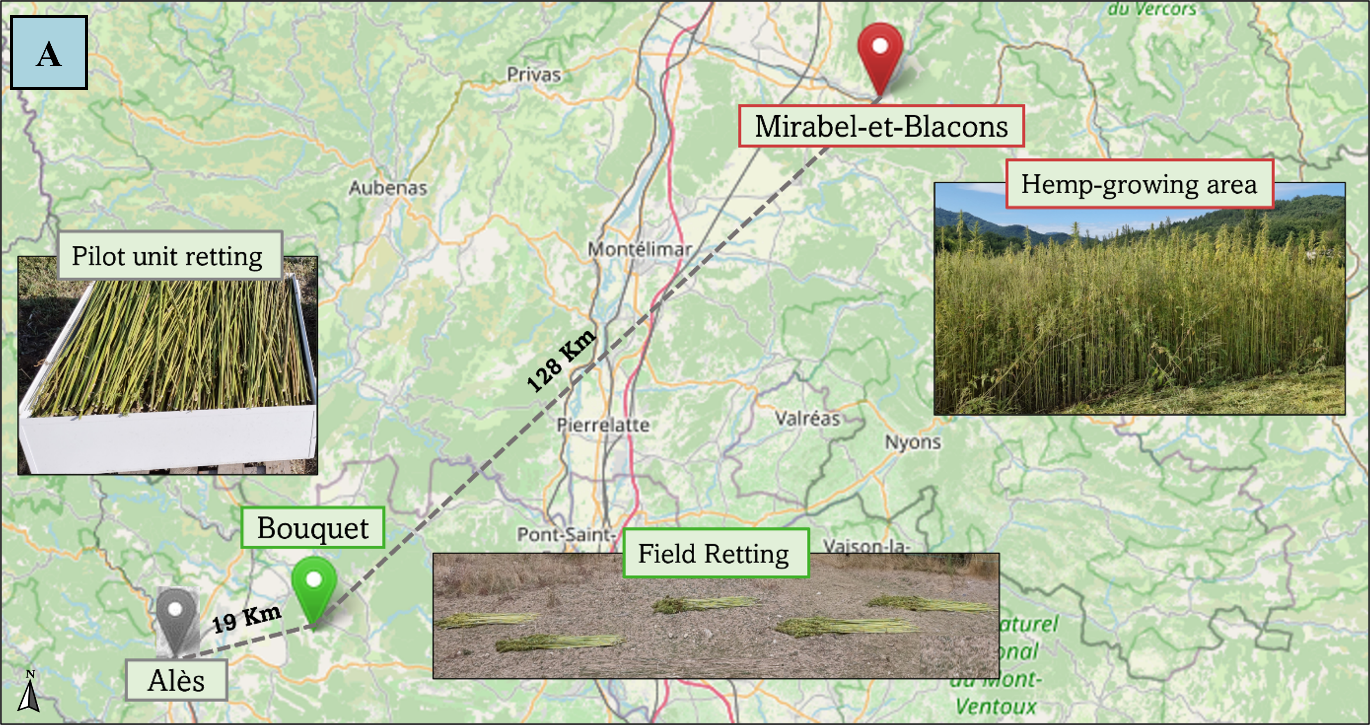
**

**
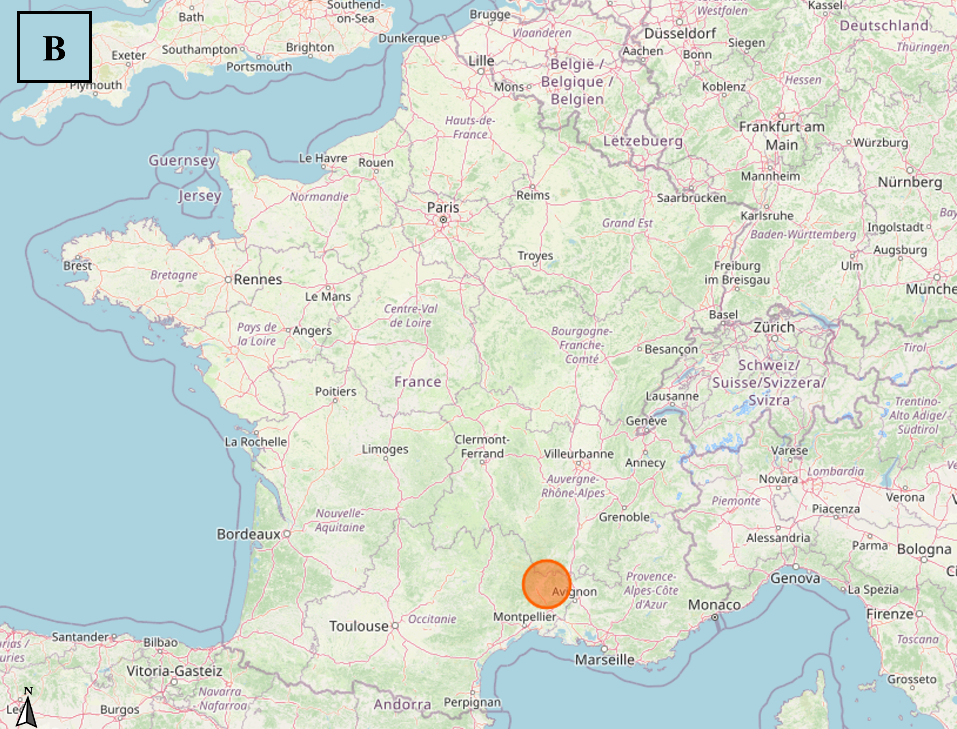
**

**Supplementary Figure 1 (A)** Sampling sites: Drôme Chanvre, Mirabel et Blacons, France (green icon), laboratory site, Alès, France (grey icon), and Mas de la Valus, Bouquet, France (red icon). **(B)** The orange circle indicates the location of the retting site relative to the country map (France). The maps are generated on OpenStreetMap.

*
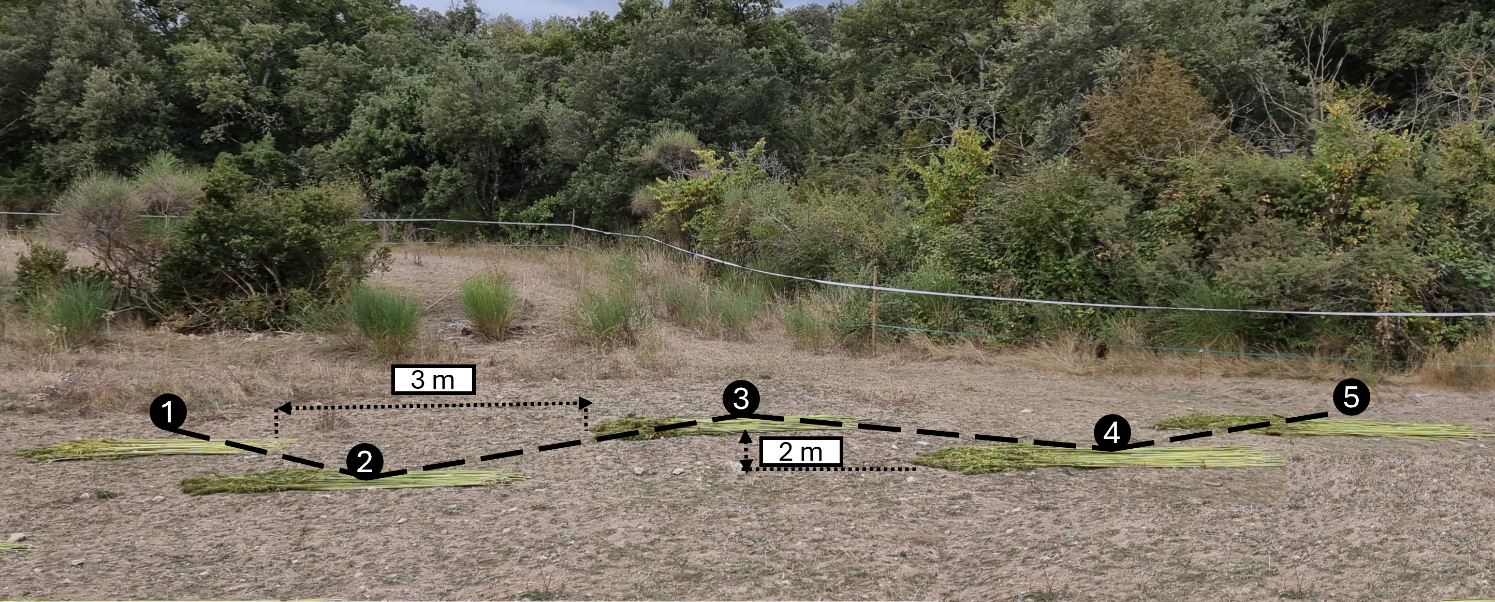
*

**Supplementary Figure 2** Experimental site during the field retting campaign. The numbers correspond to the distance (in meters) between swaths.


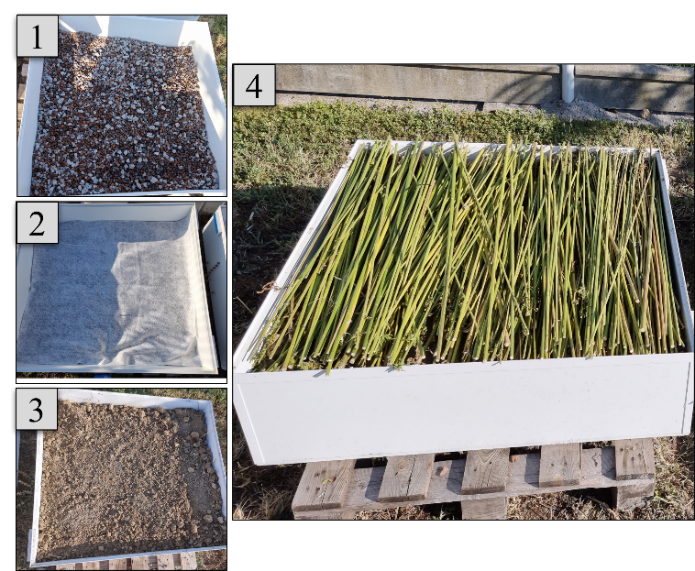


**Supplementary Figure 3** Photograph of the pilot unit illustrating the laboratory-scale retting process. The pilot unit is filled with gravel/pozzolan (10 cm high) (1) covered by a layer of geotextile (2), 15 cm of soil (3), and the hemp stems (4).


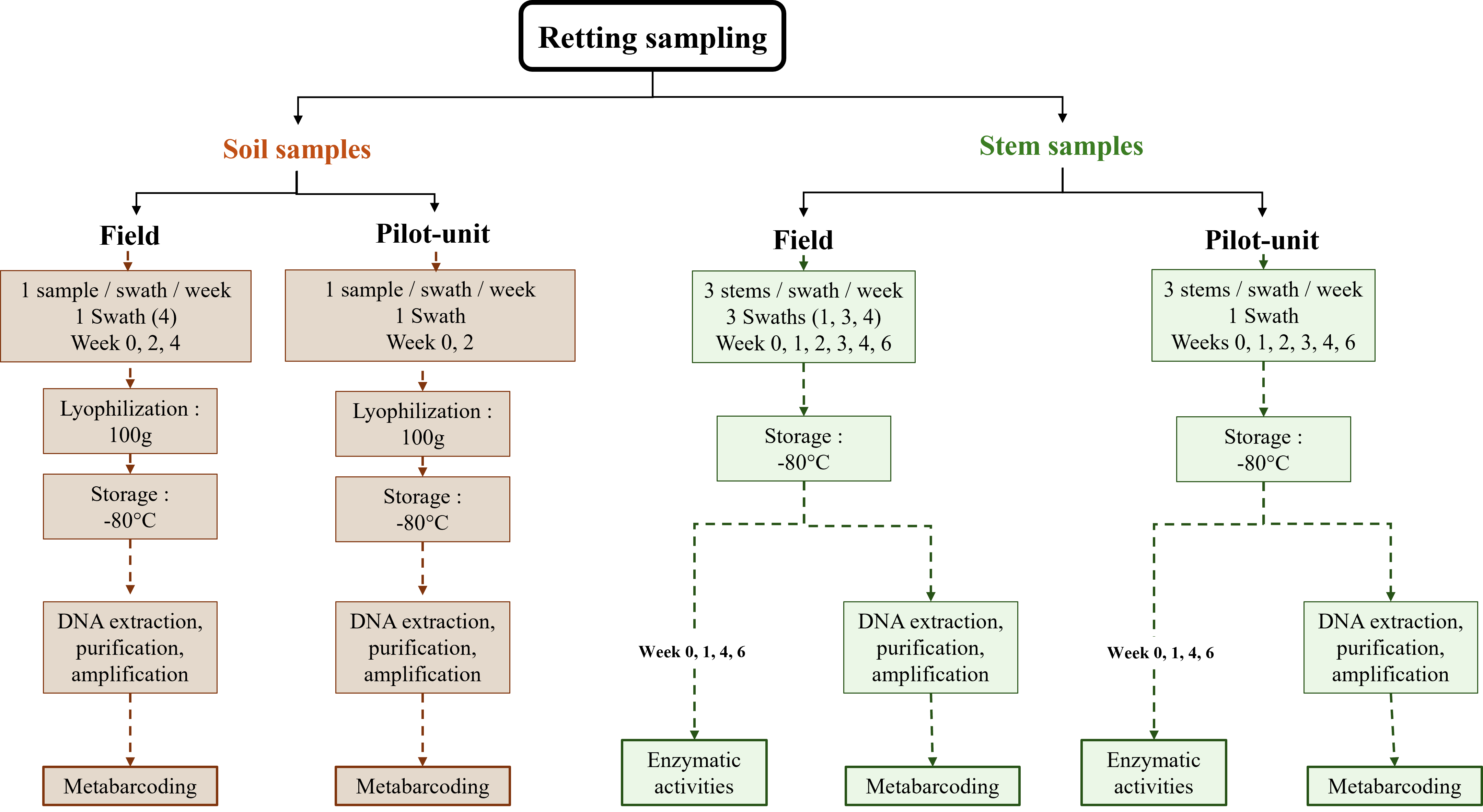


**Supplementary Figure 4** Sampling plan used during the retting campaign in the field and in the pilot unit.

**
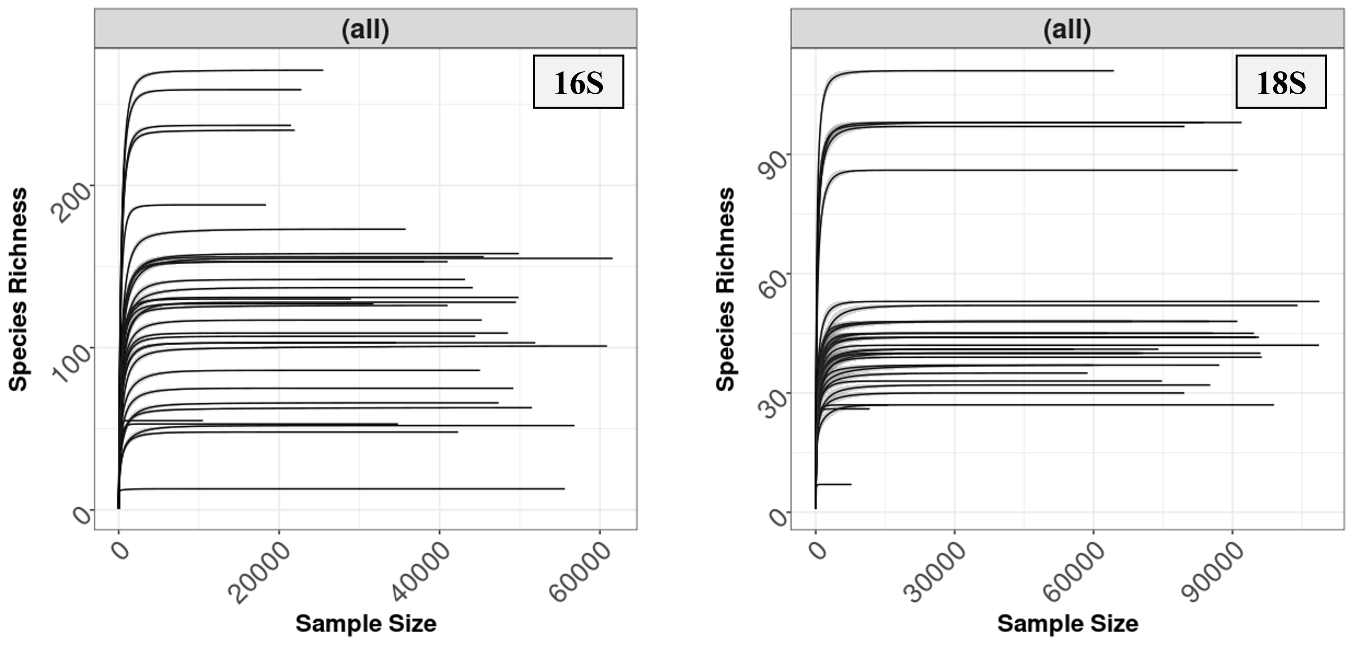
**

**Supplementary Figure 5** Rarefaction curves corresponding to bacterial (16S) and fungal (18S) domains for all samples.


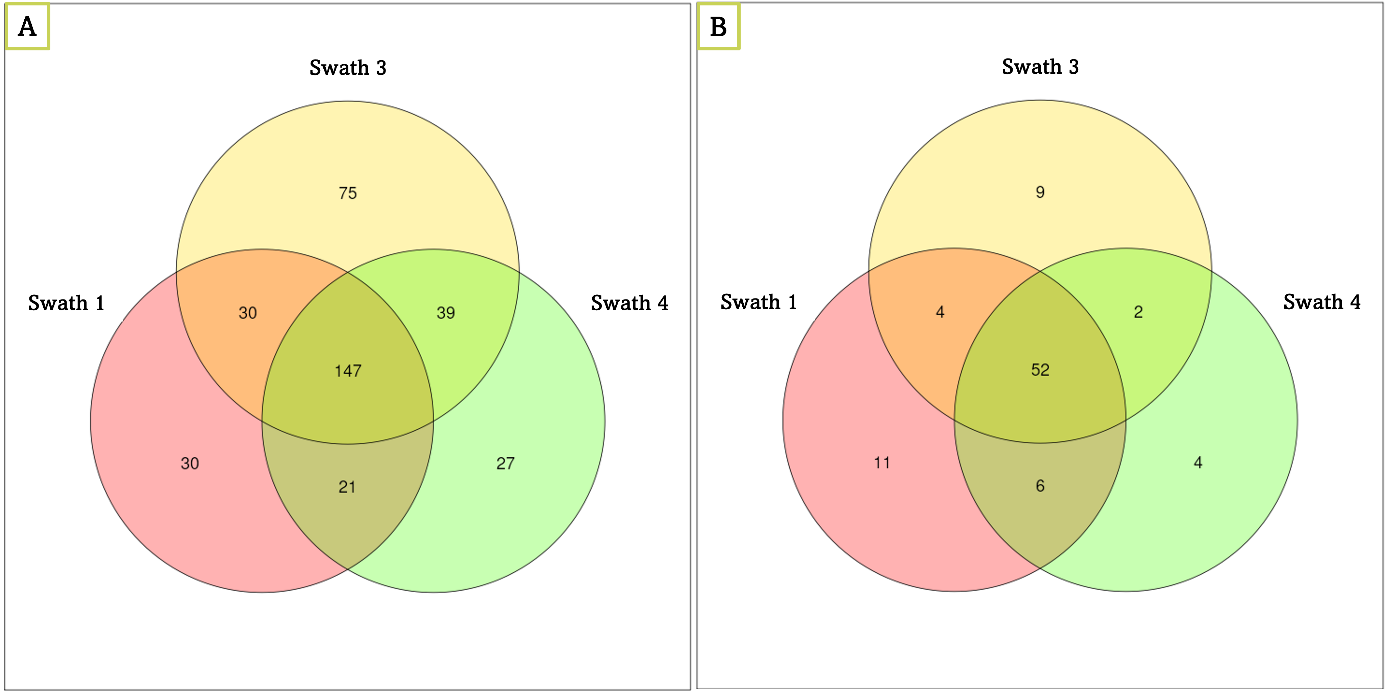


**Supplementary Figure 6** Venn Diagram illustrating the shared bacterial **(A)** and fungal **(B)** ASV among swaths 1, 3, and 4 retted in the field.


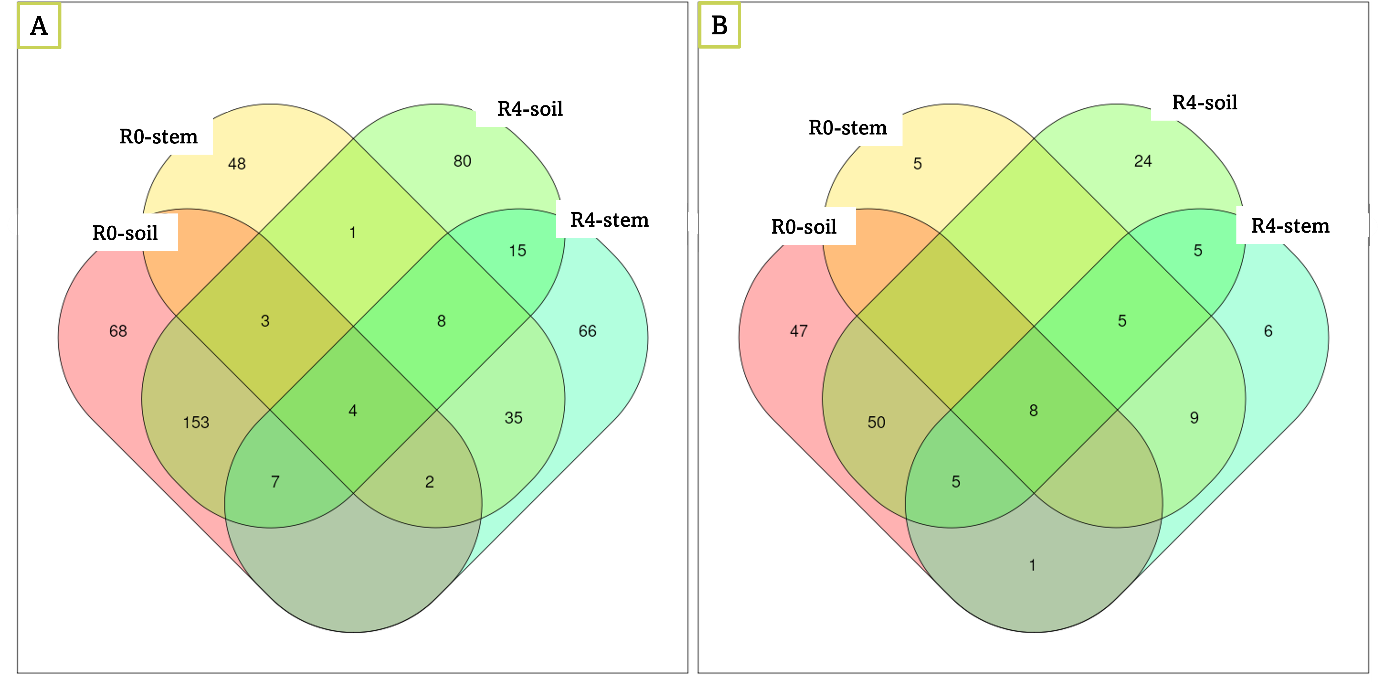
 **Supplementary Figure 7** Venn diagram of all common bacterial **(A)** and fungal **(B)** ASV between soil and stem samples (R0 and R4) belonging to the swath 4 retted in the field.


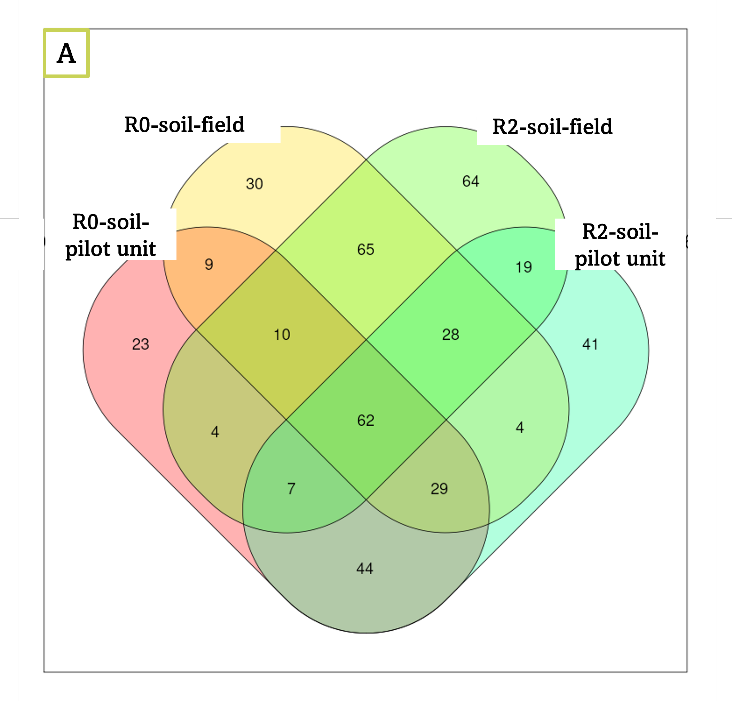

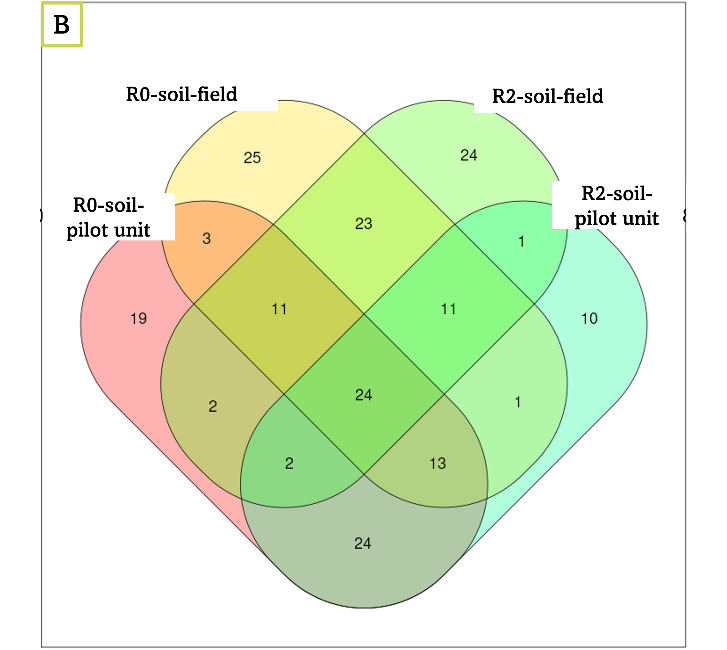


**Supplementary Figure 8** Venn diagram of all common bacterial **(A)** and fungal **(B)** ASV between soil field and soil of the pilot unit samples (R0 and R2).

**Supplementary Figure 9** Principal coordinates analysis (PCoA) of bacterial (16S) **(A)** and fungal (18S) **(B)** community structure for field and pilot unit stem and soil samples.

**Supplementary Figure 10** Bacterial relative abundance at phylum **(A)** and class **(B)** levels in unretted and retted stem samples (swaths 1, 3, and 4) in the field over time. R0: unretted samples, R1, R2, R3, R4, and R6 correspond to retted samples after 1, 2, 3, 4, and 6 weeks of retting.

**Supplementary Figure 11** Fungal relative abundance at phylum **(A)** and class **(B)** levels in unretted and retted stem samples (swaths 1, 3, and 4) in the field over time. R0: unretted samples, R1, R2, R3, R4, and R6 correspond to retted samples after 1, 2, 3, 4, and 6 weeks of retting.

**Supplementary Figure 12** Heatmaps of predicted bacterial hydrolytic enzymes present in each swath of hemp field stem samples (swaths 1, 3, and 4) and generated by PICRUSt software. **(A)** predicted cellulolytic enzymes; **(B)** predicted hemicellulolytic enzymes, and **(C)** predicted pectinolytic enzymes. Enzyme predictions are color-coded based on their raw abundance: the transition from blue through white to red reflects an increasing scale of enzyme occurrence from the lowest to the highest occurrence.
